# Left ventricular reverse remodelling after mitral transcatheter edge-to-edge repair: results from the EXPANDed studies

**DOI:** 10.1093/eschf/xvag081

**Published:** 2026-03-16

**Authors:** Mirjam Keßler, Gilbert H L Tang, Sébastien Deferm, Rodrigo Estevez-Loureiro, Wolfgang Rottbauer, Federico M Asch, Jose L Zamorano, Janani Aiyer, Rong Huang, Evelio Rodriguez, Saibal Kar, Francesco Maisano, Ralph Stephan von Bardeleben

**Affiliations:** Ulm University Heart Center, University of Ulm, Albert-Einstein-Allee 23, 89081 Ulm, Germany; Department of Cardiovascular Surgery, Mount Sinai Hospital, NewYork, NY, USA; Department of Cardiology, Hospital Oost-Limburg, Genk, Belgium; Interventional Cardiology, Hospital Alvaro Cunqueiro, Vigo, Pontevedra, Spain; Ulm University Heart Center, University of Ulm, Albert-Einstein-Allee 23, 89081 Ulm, Germany; Cardiovascular Core Laboratories, MedStar Health Research Institute, Washington, DC, USA; Department of Cardiology, Hospital Ramon y Cajal, Madrid, Spain; Clinical Affairs, Abbott Structural Heart, Santa Clara, CA, USA; Clinical Affairs, Abbott Structural Heart, Santa Clara, CA, USA; Department of Cardiac Surgery, Ascension Saint Thomas, Nashville, TN, USA; Los Robles Regional Medical Center, HCA Healthcare, Thousand Oaks, CA, USA; Department of Structural Heart Disease Interventions, San Raffaele University Hospital, Milan, Italy; Center of Structural Heart Disease Interventions, Heart Valve Center, University Medical Center of Johannes Gutenberg University, Mainz, Germany

**Keywords:** Mitral regurgitation, Mitral valve repair, Transcatheter edge-to-edge repair (TEER), Left ventricular reverse remodelling, Primary mitral regurgitation, Secondary mitral regurgitation

## Abstract

**Background and Aims:**

Left ventricular reverse remodelling (LVRR) is a key objective of contemporary heart failure (HF) therapies and is characterized by reversal of left ventricular (LV) dilation and dysfunction.

**Objectives:**

To report the incidence and clinical impact of early (30-day) LVRR patients with primary (PMR) and secondary mitral regurgitation (SMR) treated with mitral transcatheter edge-to-edge repair (M-TEER), and to identify independent associations with early LVRR.

**Methods:**

The EXPANDed cohort includes 2205 patients treated with M-TEER from the EXPAND and EXPAND G4 studies. Patients were classified as having early LVRR if they demonstrated a >10% reduction in LV dimension or volume from baseline to 30 days. All LV measurements were assessed by independent echocardiographic core laboratories.

**Results:**

Among 527 SMR patients, 338 patients (64.1%) experienced early LVRR after M-TEER. At 1 year, SMR patients with early LVRR had significantly lower rates of death or HF hospitalizations compared with those without (early LVRR: 24.7% vs no early LVRR: 35.9%, *P* = .009), despite similar MR reduction (MR mild or less ≥93% in both groups) and comparable improvements in functional status (NYHA ≤II ≥78%) and quality of life (∼20-points improvement per KCCQ-OS). Independent associations with early LVRR included hypertension [odds ratio (OR) = 1.96, *P* = .004], absence of prior cardiac surgeries (OR = 0.51, *P* = .002), and smaller LV end-systolic volume (OR = 0.81, *P* = .002).

Among 536 PMR patients, 391 (73.0%) experienced early LVRR at 30 days. At 1 year, PMR patients with early LVRR group had similar clinical (composite all-cause mortality or HF hospitalization: early LVRR: 14.5% vs no early LVRR: 17.1%, *P* = .47) and symptomatic outcomes (≥83% NYHA ≤II; ∼19-point improvement per KCCQ-OS) compared with those without. However, among PMR patients with dilated ventricles, early LVRR group was associated with significantly lower all-cause mortality (early LVRR: 3.8%, no Early LVRR: 14.0%, *P* = .028).

**Conclusions:**

Regardless of aetiology, most patients experienced early LVRR after M-TEER with significant MR reduction and symptom relief. In SMR patients, early LVRR was associated with lower rates of HF hospitalization and death.

## Graphical Abstract

**Graphical Abstract:**
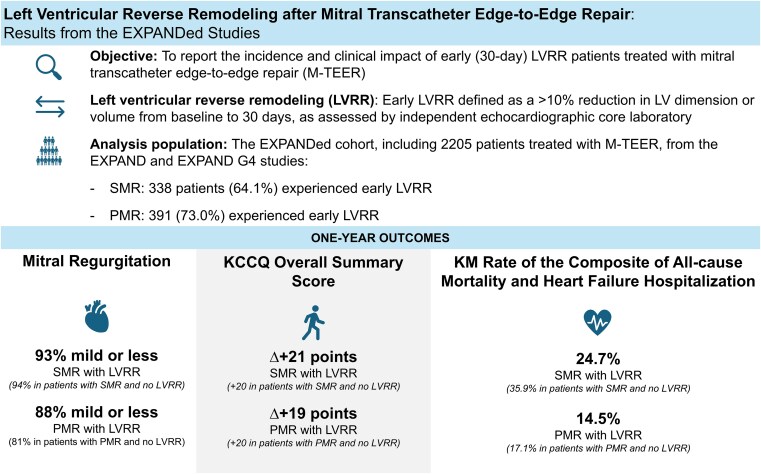

## Introduction

Adverse left ventricular (LV) remodelling or enlargement can manifest as a precursor to secondary mitral regurgitation (SMR) or can be a result of primary MR (PMR).^1,2^ This can trigger compensatory cardiac adaptations that lead to eccentric hypertrophy and enlargement of the LV chamber causing papillary muscle displacement, leaflet tethering, and impaired coaptation of mitral valve leaflets.^3,4^ The reversal of adverse remodelling from intervention can result in the desirable reduction in chamber volumes and restoration of the normal myocardium structure and function known as LV reverse remodelling (LVRR).^5^ LVRR soon after treatment is a crucial therapeutic goal associated with improved cardiac outcomes and prognosis.^6–8^

In SMR patients, the first line of treatment is guideline-directed medical therapy (GDMT), and while GDMT improves LV function, the mechanism and durability of these improvements remain unclear.^9–12^ In PMR patients, surgical mitral valve repair is recommended and has been associated with a phased LVRR process through LV dimension reduction and subsequent LV systolic function improvement.^9,13,14^ However, surgery might not be preferable in elderly patients or those considered high-risk or inoperable.^15^ Regardless of MR aetiology, residual MR severity is associated with the extent of ventricular remodelling.^16,17^

Mitral transcatheter edge-to-edge repair (M-TEER) with the MitraClip device has been used to treat MR safely and effectively in over 200 000 patients worldwide. Patients with PMR and SMR treated with the MitraClip system experience significant reductions in LV dimensions and volumes accompanied by improvements in LV ejection fraction (LVEF).^18–21^ Studies also suggest that an effective reduction in volume overload burden and ventricular stress due to MR reduction with M-TEER may result in LVRR soon after the procedure.

The objective of this analysis is to report the prevalence and impact of early (30-day) LVRR in patients with SMR and PMR undergoing MitraClip intervention and to identify independent associations in patients with early LVRR from the post-market EXPANDed studies.

## Methods

### Study design and patient cohort

The EXPAND and EXPAND G4 studies were initiated to evaluate real-world clinical experience and outcomes associated with the use of the third-generation and fourth-generation MitraClip Systems, respectively. EXPANDed is a pooled, patient-level cohort of patients from the EXPAND and EXPAND G4 studies. A total of 2205 patients in the EXPANDed cohort with PMR or SMR were enrolled at 91 centres in the USA, Europe, Canada, Israel, Saudi Arabia, and Japan from 2018 to 2022. Follow-up visits occurred at discharge, 30 days, and 1 year. All patients included in the study provided written informed consent and met the eligibility criteria according to protocol and local recommendations for use. These studies were approved by the applicable local or central ethics committees and competent authorities of participating countries and complied with the good clinical practice standards in the Declaration of Helsinki. Other study details have been previously published.^22–24^

### Echocardiographic assessment

Transthoracic and transoesophageal echocardiograms were performed at baseline and follow-up visits and assessed by an independent echocardiographic core lab (ECL). MR severity and aetiology for all EXPANDed patients were evaluated by MedStar Health Research Institute (Washington DC, USA) following the American Society of Echocardiography guidelines for ECLs. The MR aetiology was determined by the ECL as either PMR or SMR, or mixed (if both were present, and neither was the dominant mechanism). In the analysis, patients with PMR were included with those with mixed aetiology. MR severity was analysed at 30 days and 1 year follow-up.^24^ Thirty-day visits ranged from 14 to 60 days post-procedure. LV parameters were analysed by Medical Research Development S.L. (Madrid, Spain) for the EXPAND cohort and by MedStar Health Research Institute for the EXPAND G4 cohort using transthoracic images.

Early LVRR was defined as a reduction higher than 10% from baseline to 30 days in any of the following parameters: left ventricular end-diastolic volume (LVEDV), left ventricular end-systolic volume (LVESV), left ventricular end-diastolic dimension (LVEDD), or left ventricular end-systolic dimension (LVESD). This definition was chosen to algin with prior remodelling studies following device therapy.^6,20,25^ Early LVRR was analysed in patients who had paired ECL-assessed LV measurements at baseline and 30 days. Patients with missing MR aetiology or lack of paired LV measurements were excluded from the analysis.

### Clinical outcomes

The key clinical outcomes measured through 1 year included all-cause mortality, heart failure (HF) hospitalizations (HFH), and the composite outcome of all-cause mortality or HFH. New York Heart Association (NYHA) functional class and Kansas City Cardiomyopathy Questionnaire overall summary (KCCQ-OS) score were also analysed at 1 year. In EXPAND, major adverse events were adjudicated by an independent clinical events committee through 30 days and all other adverse events through 1 year were site reported. In EXPAND G4, all adverse events were site reported.

### Statistical analysis

Categorical variables are reported as percentages of available data and were compared using Fisher’s exact or chi-square tests. Bowker’s test was applied for paired nominal data. Continuous variables are presented as mean ± standard deviation unless otherwise specified and were compared using analysis of variance or the Kruskal–Wallis test for nonparametric data. Student’s *t*-test was used for paired continuous data analysis. HFH was analysed as time to first HFH event; time-to-event analyses considered only the initial HFH per patient, and patients without HFH were censored at their last known event-free date. All-cause mortality, HFH, and composite all-cause mortality or HFH were analysed using the Kaplan–Meier (KM) method with 95% confidence intervals and *P* values from log-rank tests reported between subgroups. Patients were censored at their last known event-free date. LVRR status (Early LVRR vs No Early LVRR) was modelled with multivariable logistic regression models. Variables that were statistically significant (prior cardiac surgeries, hypertension, CRT/CRT-D/ICD/permanent pacemaker, LVEF, coaptation depth, anterior posterior diastolic annular dimension at discharge) in univariate analysis were included as candidate associations for models. To assess whether the association between LVRR and the composite outcome of 1-year death or HFH differed by aetiology, we fit a Cox proportional hazards model including LVRR, aetiology, and their interaction term, adjusting for prespecified covariates. The interaction was evaluated using the Wald test. Two-sided *P* values <.05 were considered statistically significant. SAS version 9.4 (SAS Institute Inc.) was used to perform statistical analyses.

## Results

### Analysis population

**Figure 1:**
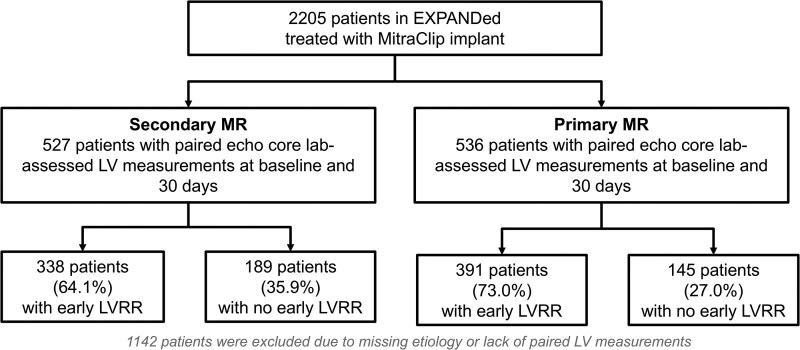
Analysis population. LVRR, left ventricular reverse remodelling; MR, mitral regurgitation

Of 2205 patients, 527 patients with SMR and 536 with PMR aetiology were available for the analysis. In 527 SMR patients, 338 patients (64.1%) experienced early LVRR while 189 patients (35.9%) did not experience early LVRR. In 536 PMR patients, 391 (73.0%) experienced early LVRR while 145 patients (27.0%) did not experience early LVRR (*Figure 1*). A total of 1142 patients were excluded due to missing MR aetiology or lack of paired LV measurements at baseline and 30 days. Of all early LVRR patients, 69.8% met the criterion through a reduction in LV volumes (i.e. LVEDV and/or LVESV). The median [IQR] for the 30-day visit echocardiogram was 37 [3; 351].

The baseline characteristics of included vs excluded patients were similar (Supplementary Table S1). In the overall cohort, 30-day LV systolic and diastolic volumes decreased from baseline by a mean of 1.5 ml (*P* = .08 compared with baseline) and 9.6 ml (*P* < .0001 compared with baseline), respectively. Similarly, 30-day LV systolic and diastolic dimensions decreased from baseline by a mean of 0.1 cm (*P* < .0001 compared with baseline) and 0.2 cm (*P* < .0001 compared with baseline), respectively.

### Secondary MR

**Figure 2:**
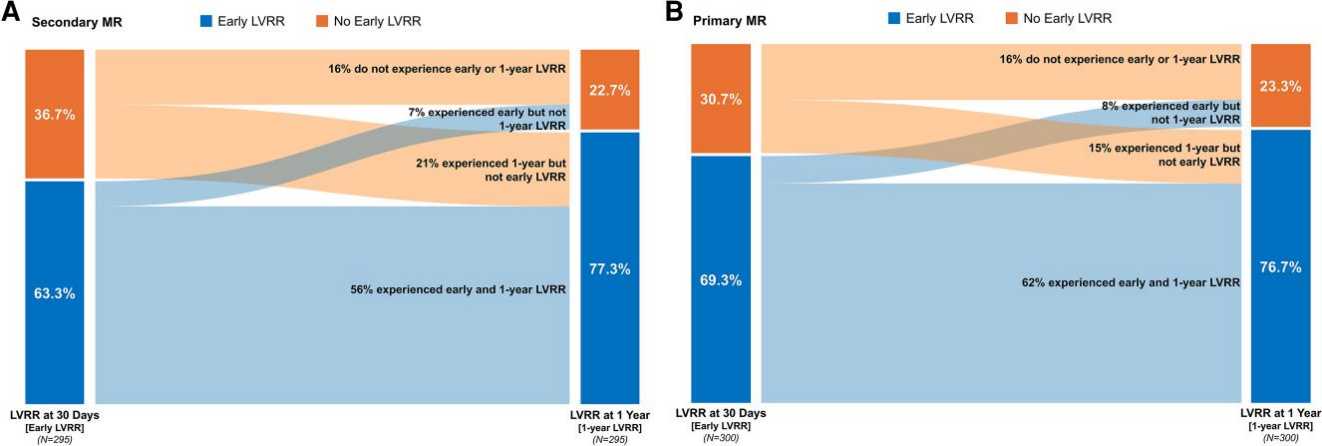
Left ventricular reverse remodelling (LVRR) through 1 year by aetiology of mitral regurgitation (MR). In patients with secondary MR (above) and primary MR (below)

In patients with SMR, 30-day LV systolic and diastolic volumes decreased from baseline by a mean of 2.0 ml (*P* = .20 compared with baseline) and 8.4 ml (*P* < .0001 compared with baseline), respectively. Similarly, 30-day LV systolic and diastolic dimensions decreased from baseline by a mean of 0.1 cm (*P* < .0001 compared with baseline) and 0.2 cm (*P* < .0001 compared with baseline), respectively. Most patients with SMR who experienced early LVRR at 30 days continued to show LVRR at 1 year (56%), while a portion of those without early LVRR underwent LVRR at 1 year (21%). A subset of patients did not experience early LVRR or LVRR at 1 year (16%) (*Figure 2*).

### Baseline characteristics in patients with secondary MR

Baseline demographics and comorbidities of SMR patients with early LVRR or no early LVRR are presented in *Table 1*. There were no significant differences in patient age, proportion of female patients, and STS risk scores for repair and replacement between the early and no early LVRR groups. Patients who experienced early LVRR had a higher proportion of hypertension than the no early LVRR group. However, patients with early LVRR were less likely to have undergone prior cardiac surgery or prior cardiac resynchronization therapy, implantable cardioverter-defibrillator, or permanent pacemaker than those with no early LVRR. Patients who experienced early LVRR had better baseline left ventricular function than those with no early LVRR, characterized by higher LV ejection fraction and smaller LV dimensions and volumes. Patients with no early LVRR were more likely to be on aldosterone antagonists, beta-blockers, diuretics, and/or cardiac medications compared with those with early LVRR. The usage of other medications at baseline was similar between both groups. (Supplementary Table S2).

**Table 1:** Baseline characteristics of patients with secondary MR

Variable	Early LVRR (*n* = 338)	No early LVRR (*n* = 189)	*P*-value
Age (years)	74.9 ± 9.5	74.1 ± 10.2	.69
Female	42.6% (144)	36.5% (69)	.17
STS repair score (%)	6.2 ± 6.1	6.5 ± 7.1	.88
STS replacement score (%)	8.2 ± 6.6	7.9 ± 5.7	.70
Hypertension	86.0% (289)	77.0% (144)	<.01
Atrial fibrillation	60.2% (203)	56.1% (105)	.40
Renal failure	36.2% (122)	37.6% (70)	.74
Myocardial infarction	28.4% (94)	29.9% (56)	.71
Prior HFH within 1 year	55.2% (171)	60.5% (107)	.26
Prior cardiac surgeries	27.2% (92)	41.3% (78)	<.001
Prior MV procedure	2.4% (8)	3.7% (7)	.42
CRT/CRT-D/ICD/permanent pacemaker	19.2% (65)	30.7% (58)	.003
Systolic pulmonary vein flow (cm/s)	41.4 ± 16.5	44.0 ± 16.3	.37
Left atrial volume (ml)	106.0 ± 58.3	109.9 ± 66.8	.60
Effective regurgitant orifice area (EROA, cm^2^)	0.3 ± 0.1	0.3 ± 0.1	.51
Regurgitant fraction (RF, %)	21.5 ± 27.7	18.2 ± 27.2	.87
Regurgitant volume (RV, ml/beat)	44.0 ± 17.5	43.6 ± 19.5	.37
A-P diastolic annular dimension (APDAD, cm)	3.5 ± 0.5	3.4 ± 0.5	.37
Left ventricular ejection fraction (LVEF, %)	41.0 ± 13.9	37.5 ± 13.4	.006
Left ventricular end systolic volume (LVESV, ml)	104.0 ± 61.1	121.2 ± 72.4	.001
Indexed LVESV (ml/m^2^)	56.2 ± 31.9	64.7 ± 37.6	.02
Left ventricular end diastolic volume (LVEDV, ml)	168.0 ± 72.4	184.4 ± 85.3	.05
Indexed LVEDV (ml/m^2^)	90.8 ± 37.1	98.6 ± 43.2	.08
Left ventricular end systolic dimension (LVESD, cm)	4.8 ± 1.1	5.1 ± 1.2	.002
Indexed LVESD (cm/m^2^)	2.6 ± 0.6	2.8 ± 0.6	.003
Left ventricular end diastolic Dimension (LVEDD, cm)	5.9 ± 0.9	6.1 ± 1.0	.008
Indexed LVEDD (cm/m^2^)	3.2 ± 0.5	3.3 ± 0.6	.04

Continuous data presented as mean ± standard deviation and compared with analysis of variance or Kruskal–Wallis test (for nonparametric data); Categorical data presented as a proportion of patients where data was provided (*n* = number of patients with available data shown) and compared with chi-squared test. Missing data was excluded.

HFH, heart failure hospitalization; LVRR, left ventricular reverse remodelling; CRT, cardiac resynchronization therapy; ICD, implantable cardiac device; MV, mitral valve; SMR, secondary mitral regurgitation; STS, Society of Thoracic Surgeons.

### MR severity in patients with secondary MR

**Figure 3:**
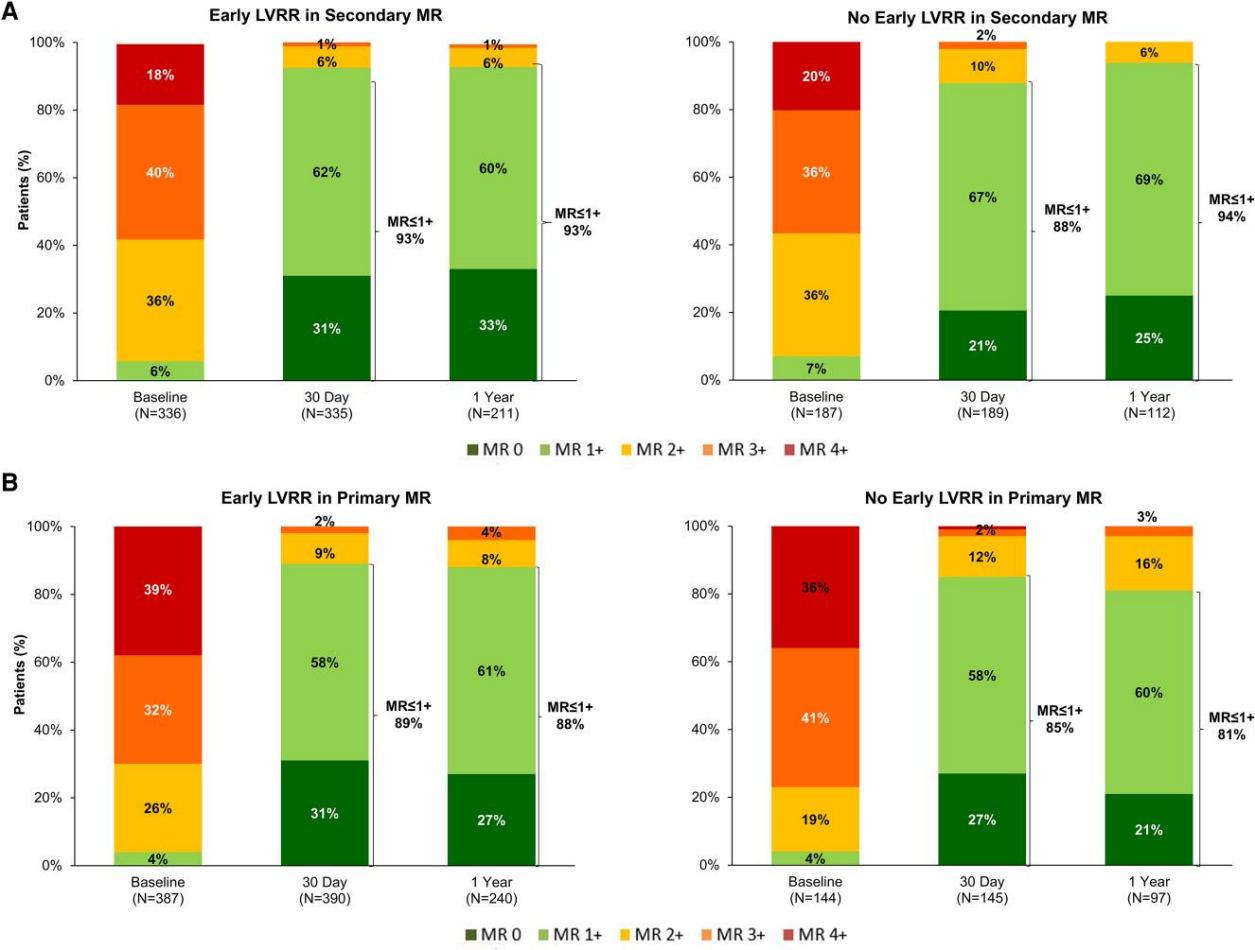
Mitral regurgitation (MR) severity through 1 year. Patients with secondary MR (above) or primary MR (below) at 30 days and 1 year by early or no left ventricular reverse remodelling (LVRR), as assessed by echocardiographic core laboratory

In patients with secondary MR, MR severity was significantly reduced at 30 days and 1 year from baseline regardless of early LVRR (*P* < .001 at 30 days and 1 year in early and no early LVRR groups compared with baseline) (*Figure 3*). After M-TEER, MR ≤1+ at 30 days and 1 year in 93% of patients with early LVRR was achieved. Among patients with no early LVRR, after M-TEER, MR ≤1+ was present in 88% at 30 days and 94% at 1 year, respectively. No significant differences between the early LVRR and the no early LVRR group were observed at baseline, 30 days, and 1 year after the procedure. The change of SMR from baseline to 1 year shows the durability of MR reduction regardless of LVRR (Supplementary Figure S1).

### Clinical outcomes in patients with secondary MR

#### All-cause mortality, HFH, and composite all-cause mortality/HFH

At 1 year, patients with early LVRR had a lower rate of all-cause mortality or HFH compared with those with no early LVRR (early LVRR: 24.7% vs no early LVRR: 35.9%, *P* = .009) (Supplementary Figure S2). One-year mortality rates for patients with early LVRR were numerically lower than those with no early LVRR without statistical significance (early LVRR: 10.5% vs no early LVRR: 15.9%, *P* = .07); however, the 1-year HFH rates were significantly lower in patients with early LVRR than those with no early LVRR (early LVRR: 18.8% vs no early LVRR: 29.1%, *P* = .01) (Supplementary Figure S3). In a sensitivity analysis using a volume-anchored definition of LVRR (>15% reduction in LVEDV or LVESV), early LVRR remained significantly associated with a lower incidence of the composite endpoint of 1-year mortality and heart failure hospitalization (22.2% vs 32.8%; log-rank *P* = .01), consistent with the primary analysis (Supplementary Figure S4). An additional sensitivity analysis applying the LVRR definition to LV dimensions and volumes indexed by body surface area similarly demonstrated a lower event rate among patients with early LVRR (24.8% vs 34.2%; log-rank *P* = .02), yielding results consistent with the primary findings (Supplementary Figure S5).

#### Functional class and quality of life

At 1 year, NYHA functional class was significantly improved at 30 days and 1 year from baseline regardless of early LVRR (Supplementary Figure S6). At baseline, 24% of patients with early LVRR had NYHA ≤II, which improved to 79% of patients having NYHA≤II at 30 days and 78% at 1 year (all *P* < .05 compared with baseline). For patients with no early LVRR, 20% had NYHA ≤II at baseline which improved to 79% at 30 days and 80% at 1 year (all *P* < .05 compared with baseline). At 1 year, regardless of early LVRR, patients experienced at least a 20-point clinically significant improvement in KCCQ-OS from baseline [KCCQ-OS change from baseline to 1 year: early LVRR: 21.1 ± 27.5, no early LVRR: 19.9 ± 26.5 (Change in KCCQ-OS from baseline to 1 year: *P* < .05)]. There were no significant differences between early and no early LVRR groups for functional capacity, quality of life, or 6-min walk distance through 1 year (Supplementary Figure S5).

#### Associations with early LVRR in patients with secondary MR

A multivariable analysis was conducted to identify the associations of patient parameters with early LVRR (Supplementary Figure S7). The univariable revealed that a higher LVEF, smaller baseline LVESD, LVEDD, LVESV, LVEDV, and the presence of prior cardiac surgeries, pacemakers, and hypertension, were associated with early LVRR (Supplementary Table S3). The multivariable analysis revealed that the presence of hypertension [odds ratio (OR) = 1.96, *P* = .004] was independently associated with early LVRR, while the presence of prior cardiac surgeries (OR = 0.51, *P* = .002) and a larger baseline LVESD (OR ampa#thinsp;0.81, *P* = .002) was associated with no early LVRR.

To assess whether the association between early LVRR and the composite outcome of 1-year death or HFH differed by aetiology, a Cox proportional hazards model was used that included LVRR, aetiology (SMR vs PMR), and their interaction term, adjusting for the prespecified covariates. Aetiology remained significantly associated with the composite outcome (adjusted HR ampa#thinsp;0.55; 95% CI: 0.36–0.84), whereas LVRR was associated with a reduced hazard (adjusted HR = 0.66; 95% CI: 0.48–0.90). The interaction term was not significant (*P*-interaction = .99), indicating no evidence that the effect of LVRR on the composite endpoint differed by aetiology.

#### Primary MR

In all patients with PMR, 30-day LV systolic and diastolic volumes decreased from baseline by a mean of 1.1 ml (*P* = .15 compared with baseline) and 10.7 ml (*P* < .0001 compared with baseline), respectively. Similarly, 30-day LV systolic and diastolic dimensions decreased from baseline by a mean of 0.2 cm (*P* < .0001 compared with baseline) and 0.3 cm (*P* < .0001 compared with baseline), respectively. In patients with PMR, 62% who showed early LVRR at 30 days continued to demonstrate LVRR at 1 year. Additionally, 15% of those without early LVRR went on to exhibit LVRR at 1 year, while 16% showed no LVRR at either time point (*Figure 2*).

### Baseline characteristics in patients with primary MR

Baseline demographics and comorbidities of PMR patients with early LVRR or no early LVRR are presented in *Table 2*. There were no significant differences in patient age, proportion of female patients, and STS risk scores for repair and replacement between the early and no early LVRR groups. There was a similar incidence of comorbidities between both groups including hypertension, atrial fibrillation, renal failure, and myocardial infarction. Patients who experienced early LVRR had a higher incidence of prior MV procedures than the no early LVRR group. Compared with secondary MR patients, PMR was associated with less dilated left ventricular dimensions and volumes and higher left ventricular ejection fraction. Patients who experienced early LVRR had larger left ventricular dimensions and volumes than those in the no early LVRR group.

**Table 2:** Baseline characteristics of patients with primary MR

Variable	Early LVRR (*n* = 391)	No early LVRR (*n* = 145)	*P*-value
Age (years)	79.5 ± 8.8	80.0 ± 8.7	.60
Female	45.3% (177)	49.7% (72)	.37
STS repair score (%)	5.0 ± 4.3	5.6 ± 6.1	.28
STS replacement score (%)	6.8 ± 5.2	7.0 ± 4.2	.70
Hypertension	75.4% (294)	80.6% (116)	.21
Atrial fibrillation	52.8% (205)	57.9% (84)	.33
Renal failure	36.2% (122)	37.6% (70)	.74
Myocardial infarction	12.1% (47)	12.6% (28)	.88
Prior HFH within 1 year	40.1% (144)	38.2% (50)	.70
Prior Cardiac Surgeries	16.6% (65)	18.6% (27)	.59
Prior MV procedure	7.7% (30)	2.8% (4)	.04
CRT/CRT-D/ICD/permanent pacemaker	5.4% (21)	2.8% (4)	.20
Systolic pulmonary vein flow (cm/s)	49.9 ± 21.5	48.7 ± 15.3	.77
Mixed MR aetiology	6.1% (24)	6.2% (9)	.98
Left atrial volume (ml)	103.5 ± 52.0	89.2 ± 43.6	.09
Effective regurgitant orifice area (EROA, cm^2^)	0.4 ± 0.2	0.4 ± 0.2	.28
Regurgitant fraction (RF, %)	15.6 ± 26.8	12.8 ± 23.5	.47
Regurgitant volume (RV, ml/beat)	56.8 ± 24.5	55.3 ± 24.1	.60
A-*P* diastolic annular dimension (APDAD, cm)	3.3 ± 0.5	3.2 ± 0.5	.24
Left ventricular ejection fraction (LVEF, %)	61.1 ± 9.9	60.3 ± 11.1	.72
Left ventricular end systolic volume (LVESV, ml)	49.1 ± 27.0	46.5 ± 30.2	.04
Indexed LVESV	27.0 ± 13.7	25.6 ± 15.2	.02
Left ventricular end diastolic volume (LVEDV, ml)	123.3 ± 45.0	111.9 ± 43.8	.004
Indexed LVEDV	68.2 ± 21.5	61.8 ± 20.6	.001
Left ventricular end systolic dimension (LVESD, cm)	3.6 ± 0.8	3.4 ± 0.8	.01
Indexed LVESD	2.0 ± 0.4	1.9 ± 0.4	.008
Left ventricular end diastolic dimension (LVEDD, cm)	5.2 ± 0.7	5.1 ± 0.7	.39
Indexed LVEDD	2.9 ± 0.4	2.9 ± 0.4	.64

Continuous data presented as mean ± standard deviation and compared with analysis of variance or Kruskal–Wallis test (for nonparametric data); Categorical data presented as a proportion of patients where data were provided (*n* = number of patients with available data shown) and compared with chi-squared test. Missing data were excluded.

HFH, heart failure hospitalization; LVRR, left ventricular reverse remodelling; CRT, cardiac resynchronization therapy; ICD, implantable cardiac device; MV, mitral valve; PMR, primary mitral regurgitation; STS, Society of Thoracic Surgeons.

### MR severity in patients with primary MR

In patients with primary MR, MR severity was significantly reduced at 30 days and 1 year from baseline regardless of early LVRR (*Figure 3*). After M-TEER, MR ≤1+ was present in 89% of patients at 30 days and 88% at 1 year (all *P* < .001 compared with baseline). Among patients with no early LVRR, after M-TEER, MR ≤1+ was present in 85% at 30 days and 81% at 1 year, respectively (all *P* < .001 compared with baseline). No significant differences between the early LVRR and the no early LVRR group were observed at baseline, 30 days, and 1 year after the procedure. The change of PMR from baseline to 1 year exhibited durability of MR reduction regardless of LVRR (Supplementary Figure S8).

### Clinical outcomes in patients with primary MR

#### All-cause mortality, HFH, and composite

At 1 year, patients with early LVRR had a similar rate of all-cause mortality or HFH compared with those with no early LVRR (early LVRR: 14.5% vs no early LVRR: 17.1%, *P* = .47) (Supplementary Figure S9). These results were consistent when the outcomes were observed with similar 1-year mortality (early LVRR: 6.1% vs no early LVRR: 10.0%, *P* = .13) and HFH rates (early LVRR: 10.9% vs no early LVRR: 11.6%, *P* = .83) (Supplementary Figure S10).

**Figure 4:**
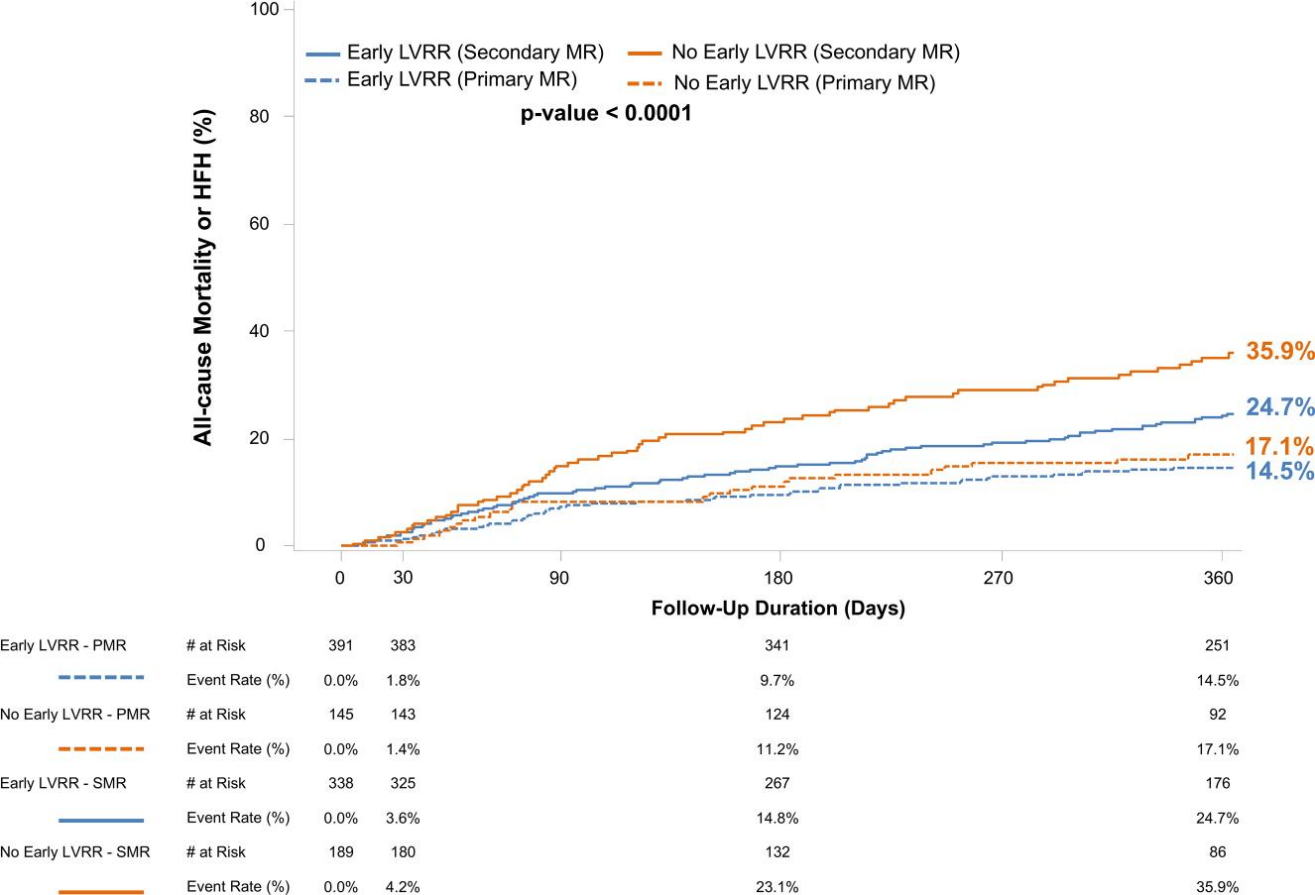
Composite of all-cause mortality or heart failure hospitalization (HFH) through 1 year by early left ventricular reverse remodelling (LVRR) and aetiology. Kaplan–Meier estimates for patients with primary mitral regurgitation (primary MR, dashed lines) and secondary MR (secondary MR, solid lines) and either early left ventricular reverse remodelling (LVRR, blue) or no early LVRR (orange). Significance by log-rank test

The composite of all-cause mortality and HFH through 1 year showed a higher event rate in SMR patients compared with PMR patients (*Figure 4*).

To evaluate the prognostic relevance of LVRR in patients with PMR, patients were stratified into cohort-derived tertiles by baseline LVEDD as an exploratory approach. Baseline LVEDD was selected as the variable of interest as it had the most complete data and was highly correlated with other LV dimensions and volumes. Patients with LVEDD ≥5.4 cm represent the third tertile, while those with LVEDD <5.4 cm comprise the first and second tertiles combined. Among PMR patients with dilated ventricles (LVEDD ≥ 5.4 cm), those who experienced early LVRR had lower mortality rates over 1 year compared with those who did not experience early LVRR (early LVRR: 3.8%, no early LVRR: 14.0%, *P* = 0.028). In contrast, among patients with non-dilated ventricles (LVEDD <5.4 cm), survival was not impacted by early LVRR (early LVRR: 7.2%, no early LVRR: 8.3%, *P* = .71). Additionally, among 177 patients with dilated ventricles, the majority underwent early LVRR [75% (132/177)] (Supplementary Figure S10).

#### Functional class and quality of life

At 1 year, NYHA functional class was significantly improved at 30 days and 1 year from baseline regardless of early LVRR (Supplementary Figure S11). At baseline, 33% of patients with early LVRR had NYHA ≤II which improved to 85% of patients having NYHA ≤II at 30 days and 83% at 1 year (all *P* <.001 compared with baseline). Among patients with no early LVRR, 36% had NYHA ≤II at baseline which improved to 90% at 30 days and 91% at 1 year (all *P* < .001 compared with baseline). At 1 year, regardless of early LVRR, patients experienced a clinically significant improvement in quality-of-life as assessed by KCCQ-OS from baseline [KCCQ-OS change from baseline to 1 year: early LVRR: 18.8 ± 24.4, no early LVRR: 20.1 ± 21.7 (Change in KCCQ-OS from baseline to 1 year: *P* < .001)]. There were no differences between early and no early LVRR groups for functional capacity, quality of life, or 6-min walk distance through 1 year (Supplementary Figure S12).

#### Associations with early LVRR in patients with primary MR

Although early LVRR did not impact clinical analyses, a multivariable analysis was conducted to identify the associations of patient parameters with early LVRR. The univariable analysis is presented in Supplementary Table S4. The multivariable analysis revealed that a larger baseline LVEDV [OR = 1.01 (95% CI: 1.00, 1.01) per ml LVEDV, *P* = 0.01] was associated with early LVRR.

## Discussion

The pathological processes of adverse LV remodelling can be observed in patients both with PMR and SMR. However, the underlying mechanism causing LV remodelling is distinct. Whereas PMR causes volume overload leading to progressive LV enlargement, SMR is a consequence of adverse LV remodelling processes in the context of HF or other cardiac pathologies. For this analysis, we hypothesized that elimination of volume overload resulting from the elimination of MR after M-TEER would induce reverse remodelling of the LV in both PMR and SMR. However, because of the distinct mechanisms behind the initial adverse remodelling for the two MR aetiologies, we analysed the occurrence and associations with LVRR after M-TEER by MitraClip in the global EXPANDed studies in SMR and PMR separately.

In SMR, 64.1% experienced early LVRR, indicating a change of >10% in LV dimensions or volumes within 30 days after M-TEER. In a post-hoc analysis of the randomized COAPT trial (including 348 patients of which 190 were treated with M-TEER, 158 were control patients) roughly half of the SMR patients experienced LVRR within 6 months.^26^ In contrast to the COAPT analysis where a definition for LVRR of 6 months was used, in the EXPANDed studies, LVRR as early as 30 days after M-TEER was analysed. In the EXPANDed studies, SMR patients with early LVRR more often had arterial hypertension but less frequently a history of cardiac rhythm implants or previous cardiac surgery. Additionally, the LV was less dilated with better LVEF compared with patients with no early LVRR. This may suggest a less advanced stage of HF in the early LVRR group. Compared with the COAPT trial, LVs of EXPANDed SMR patients seem to be less severely diseased: In the COAPT trial, LV volumes were even larger (198 ± 72 ml in COAPT vs 168 ± 74 ml in the EXPANDed early LVRR vs 184 ± 85 ml in the EXPANDed no early LVRR). Likewise, LVEF was even more reduced in COAPT (31% vs 41% vs 38%, respectively). M-TEER by MitraClip was equally effective in SMR patients in the EXPANDed studies in the early LVRR and no early LVRR groups.

Symptom control measured by NYHA class and KCCQ-OS score improved significantly and comparably regardless of early LVRR in the SMR patients in the EXPANDed studies. Within the 1-year follow-up after M-TEER, SMR patients with early LVRR had significantly lower rates of the composite of HFH or mortality (25% vs 36%, *P* = .009). This difference was mainly driven by significant differences in the HFH rate (19% vs 29%, *P* = .01) but also a trend towards lower mortality in the early LVRR group (11% vs 16%, *P* = .07). This beneficial impact of LVRR on survival and HFH in SMR patients in the EXPANDed studies further supports previous studies.^20,26,27^

We aimed to identify associations with early LVRR in SMR patients and found the presence of arterial hypertension (OR 1.96, *P* = .004), absence of previous cardiac surgery (OR = 0.51, *P* = .002), and smaller baseline LVESD (OR 0.81, *P* = .002) were independently associated with early LVRR. Interestingly, MR severity after M-TEER was not associated with early LVRR. This suggested association, albeit weakly predictive, is in line with the recent COAPT subanalysis^26^ but in some more historic studies, a trend towards lower procedural success had been observed in patients without LVRR.^20,27^ However, recent improvements in procedural success rates, operator experience, device technical abilities, and a postprocedural MR grade have rendered residual MR as a less common influential factor in procedural outcome.^22,23^

The association of LV dimensions and volumes with the development of LVRR has been described before for M-TEER patients.^20^ More advanced HF stages seem to be less susceptible to LVRR. Potentially, this is not only a matter of LV size but also of the irreversible replacement of myocardium with scar tissue or fibrosis, as it has been also hypothesized previously^20,28^ This potential irreversible damage might even be reflected by a history of cardiac surgeries, as these often are necessary in the natural course of HF, in particular in ischaemic HF in which scars and fibrosis are a mandatory feature. Contrarily, the presence of arterial hypertension was an association with early LVRR in the EXPANDed studies. This interaction of blood pressure with LVRR has previously been described for HFrEF^29,30^ and suggests that ‘preserved blood pressure’ may be a potentially beneficial prognostic factor for LVRR in HF and SMR; however, larger studies with increased associative power are needed.

Compared with SMR, the data on LVRR after M-TEER for PMR is scarce. In the EXPANDed studies, 73% of the PMR patients experienced early LVRR after M-TEER. In PMR patients, early LVRR was more often observed with dilated LV dimensions and volumes, which at first sight might seem contradictory to the findings in SMR patients. However, not surprisingly, compared with SMR patients, dilation of the LV was less pronounced in the PMR cohort (123 ml in the early LVRR-PMR patients, 111 ml in the no early LVRR-PMR patients, compared with 168 ml/184 ml in the SMR cohort, respectively). Naturally, PMR patients with no or only mild dilation of the LV did not experience LVRR.

As also observed for SMR, PMR patients experienced significant MR reduction along with symptomatic relief reflected by NYHA class or KCCQ-OS score irrespective of early LVRR. Interestingly, no difference in the composite of mortality or HFH was found in PMR patients regardless of early LVRR (14% vs 18%, *P* =.47). This finding suggests two contradictory hypotheses. For one, early LVRR in PMR patients might not be as important for their survival and HFH as observed in SMR patients. This is underlined by the different pathomechanism of PMR, where LV dilation is caused by volume overload due to MR and not a genuine ventricular disease like in SMR. For the second hypothesis, these results may demonstrate that even PMR patients with dilated LVs, and potentially indicative of ‘late’ M-TEER, commonly experience early LVRR and have comparable clinical outcomes and symptom relief.

### Limitations

This analysis has several limitations. Only 48.2% of 2205 patients in the EXPANDed studies were included in the early LVRR analysis. Main reasons for exclusion were the unavailability of LV dimensions or volumes at baseline or 30 days or that MR aetiology could not be classified. 3D echocardiography to determine LV volumes would be the preferable method instead of 2D echocardiography, which was used for this ECL analysis in the EXPANDed studies. Furthermore, patients that died within 30 days after the procedure were also not available for early LVRR analysis, which might lead to a bias of the results of this analysis, although other studies have assessed LVRR^20,26,27^ at later timepoints (e.g. 6 months or 12 months after M-TEER) this with similar concerns. The presented follow-up period of 12 months might be too short to detect the influence of early LVRR on long-term clinical outcomes, in particular in the PMR subgroup. Furthermore, as early LVRR was defined at 30 days, it cannot be excluded that patients in the no early LVRR group could experience LVRR at a later timepoint upon longer follow-up. Data on GDMT were limited to proportion of patients on GDMT (i.e. titration was not collected) and followed per real-world adherence. Finally, additional studies are needed to assess how LVRR may translate to changes in exercise capacity or biomarkers, which are not reported here.

## Conclusion

Most patients in EXPANDed experience early LVRR after M-TEER by MitraClip regardless of MR aetiology. In both MR aetiologies, M-TEER led to significant MR reduction along with relevant symptom relief regardless of the occurrence of early LVRR. In SMR patients, early LVRR was additionally associated with lower probability of HFH and mortality. Early LVRR in PMR patients occurred more often in dilated left LVs, indicative of relevant volume overload, but was not associated with a worse clinical outcome.

## Supplementary Material

xvag081_Supplementary_Data

## Declarations

### Data Availability

No data were generated or analysed for this manuscript.

### Pre-registered Clinical Trial Number

None supplied.
