# Supplementary material for: Left ventricular reverse remodelling after mitral transcatheter edge-to-edge repair: results from the EXPANDed studies
Source: ESC Heart Fail. 2026 Mar 16;13(3):xvag081. doi: 10.1093/eschf/xvag081 (PMC13282899; doi:10.1093/eschf/xvag081)
Supplement: xvag081_Supplementary_Data [file xvag081_supplementary_data.docx]

**SUPPLEMENTAL MATERIALS**

| **Supplemental Figures** | **Page(s)** |
| --- | --- |
| **Supplemental Figure 1.** Paired mitral regurgitation (MR) severity through 1 year by early left ventricular reverse remodeling (LVRR) in patients with secondary MR. | **3** |
| **Supplemental Figure 2.** Composite all-cause mortality or heart failure hospitalization (HFH) through 1 year in patients with secondary mitral regurgitation (MR). | **4** |
| **Supplemental Figure 3.** All-cause mortality and heart failure hospitalization (HFH) through 1 year in patients with secondary mitral regurgitation. | **5** |
| **Supplemental Figure 4.** Composite all-cause mortality or heart failure hospitalization (HFH) through 1 year in patients with secondary mitral regurgitation (MR) with a volume anchored and stricter definition for early LVRR. | **6** |
| **Supplemental Figure 5.** Composite all-cause mortality or heart failure hospitalization (HFH) through 1 year in patients with secondary mitral regurgitation (MR) applying the LVRR definition with indexed LV dimensions and volumes (indexed by body surface area). | **7** |
| **Supplemental Figure 6.** Functional and symptomatic outcomes through 1 year in patients with secondary mitral regurgitation (MR). | **8-9** |
| **Supplemental Figure 7**. Associations with early left ventricular reverse remodeling (LVRR) in patients with secondary mitral regurgitation. | **10** |
| **Supplemental Figure 8.** Paired mitral regurgitation (MR) severity through 1 year by early left ventricular reverse remodeling (LVRR) in patients with primary MR. | **11** |
| **Supplemental Figure 9.** Composite all-cause mortality or heart failure hospitalization (HFH) through 1 year in patients with primary mitral regurgitation (MR). | **12** |
| **Supplemental Figure 10.** All-cause mortality and heart failure hospitalization (HFH) through 1 year in patients with primary mitral regurgitation. | **13** |
| **Supplemental Figure 11.** All-cause mortality by baseline left ventricular end diastolic dimension (LVEDD) and presence of early or not early left ventricular reverse remodeling (LVRR) in patients with primary mitral regurgitation. | **14** |
| **Supplemental Figure 12.** Functional and symptomatic outcomes through 1 year in patients with primary mitral regurgitation (MR). | **15** |

| **Supplemental Tables** | **Page(s)** |
| --- | --- |
| **Supplemental Table 1.** Baseline characteristics of patients excluded from analysis due to missing paired left ventricular (LV) measurements. | **16** |
| **Supplemental Table 2.** Baseline medication usage in patients with secondary mitral regurgitation. | **17** |
| **Supplemental Table 3.** Univariable analysis with early LVRR in patients with secondary mitral regurgitation. | **18-19** |
| **Supplemental Table 4.** Univariable analysis with early LVRR in patients with primary mitral regurgitation. | **20-21** |


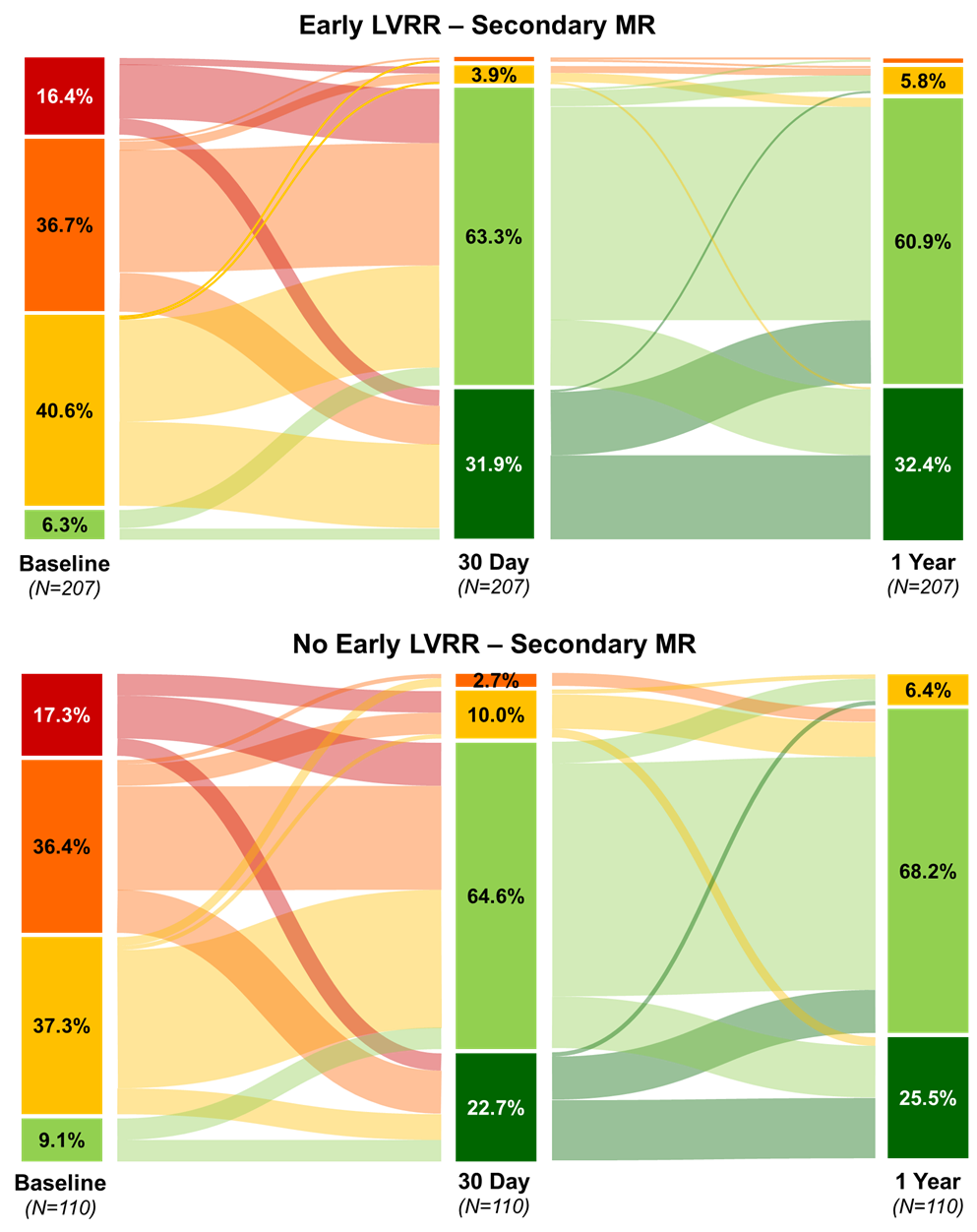


**Supplemental Figure 1. Paired mitral regurgitation (MR) severity through 1 year by early left ventricular reverse remodeling (LVRR) in patients with secondary MR.**

**
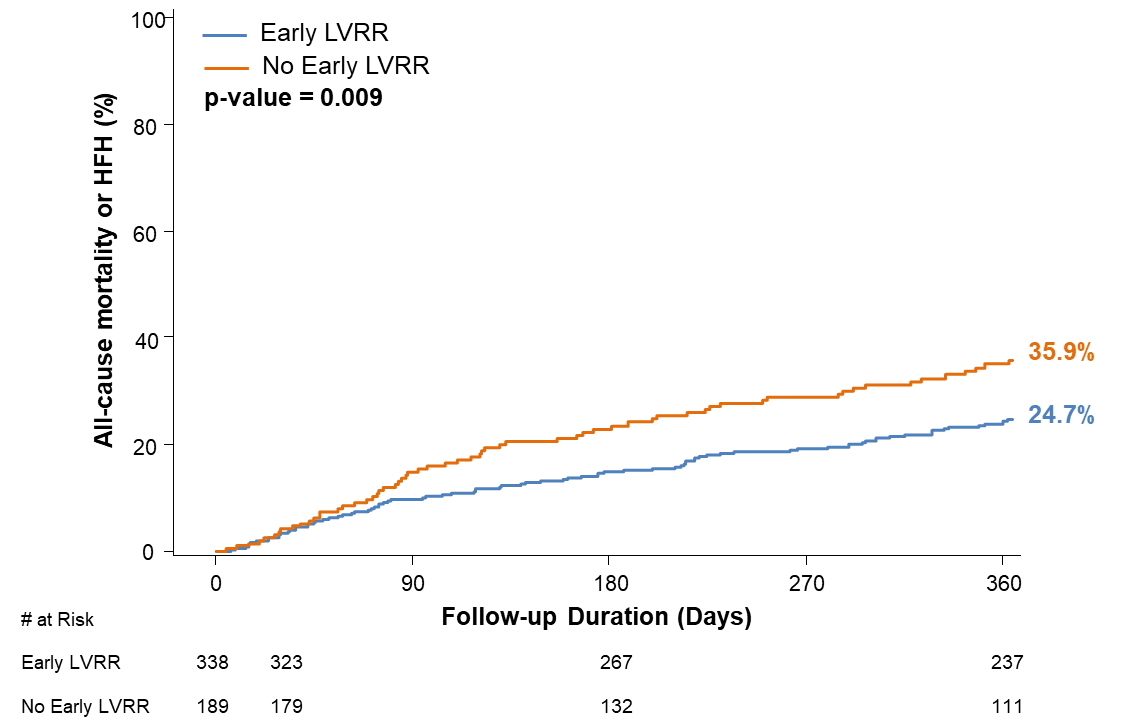
**

**Supplemental Figure 2. Composite all-cause mortality or heart failure hospitalization (HFH) through 1 year in patients with secondary mitral regurgitation (MR).** Kaplan-Meier estimate for patients with early left ventricular reverse remodeling (LVRR, blue) and no early LVRR (orange). Significance by log-rank test.

**
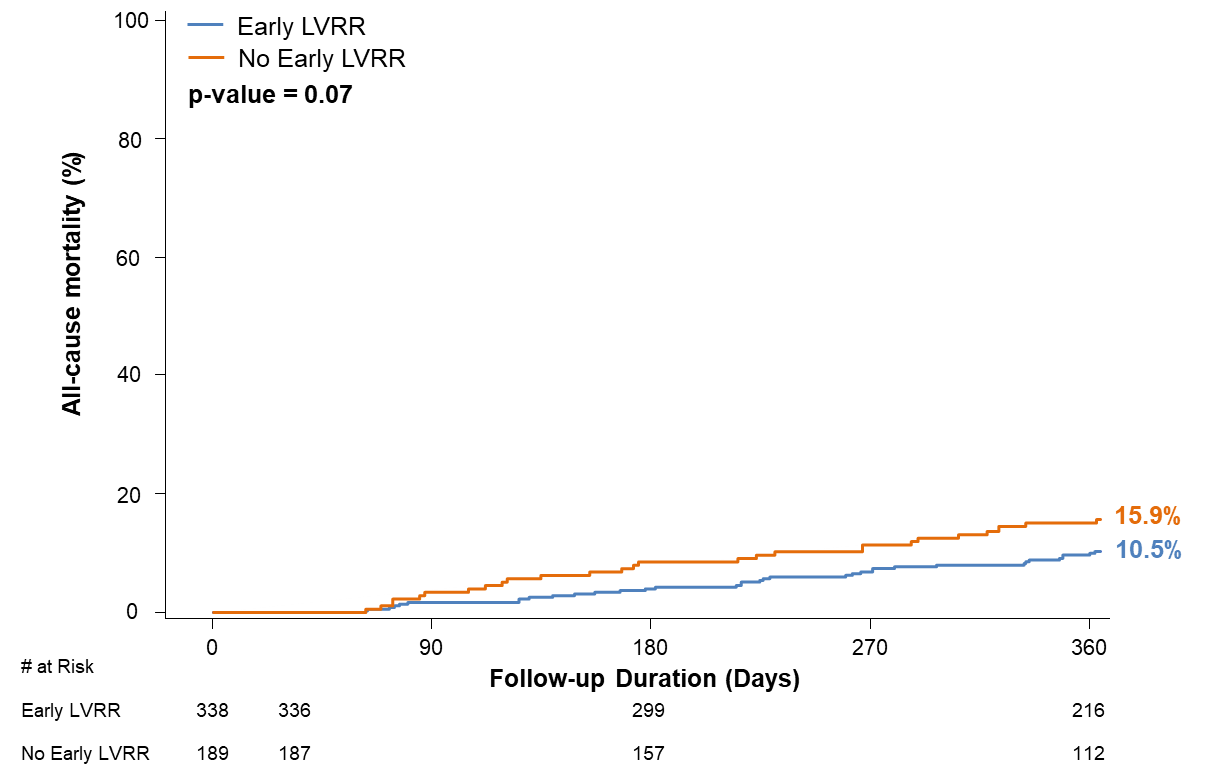
**

**
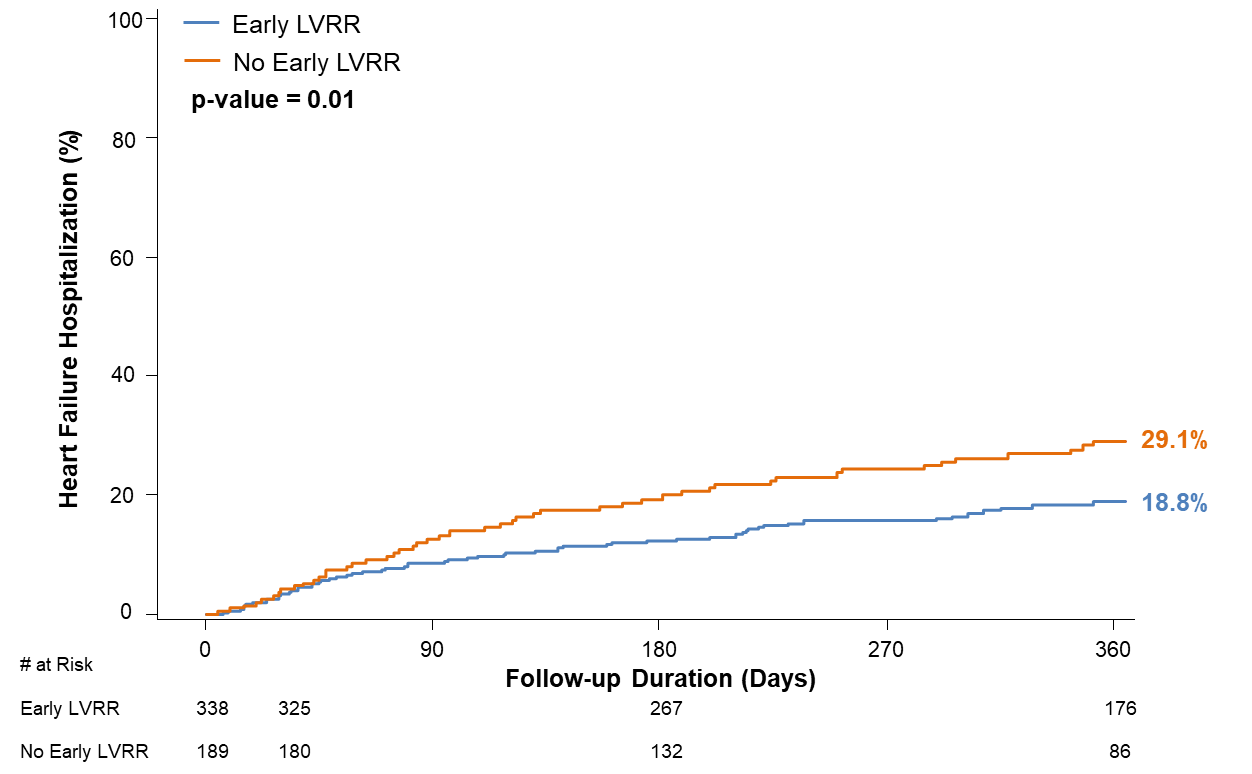
**

**Supplemental Figure 3. All-cause mortality and heart failure hospitalization (HFH) through 1 year in patients with secondary mitral regurgitation.** Kaplan-Meier estimate of all-cause mortality in patients with secondary mitral regurgitation in either early left ventricular reverse remodeling (LVRR, blue) or no early LVRR (orange) groups. Significance by log-rank test.


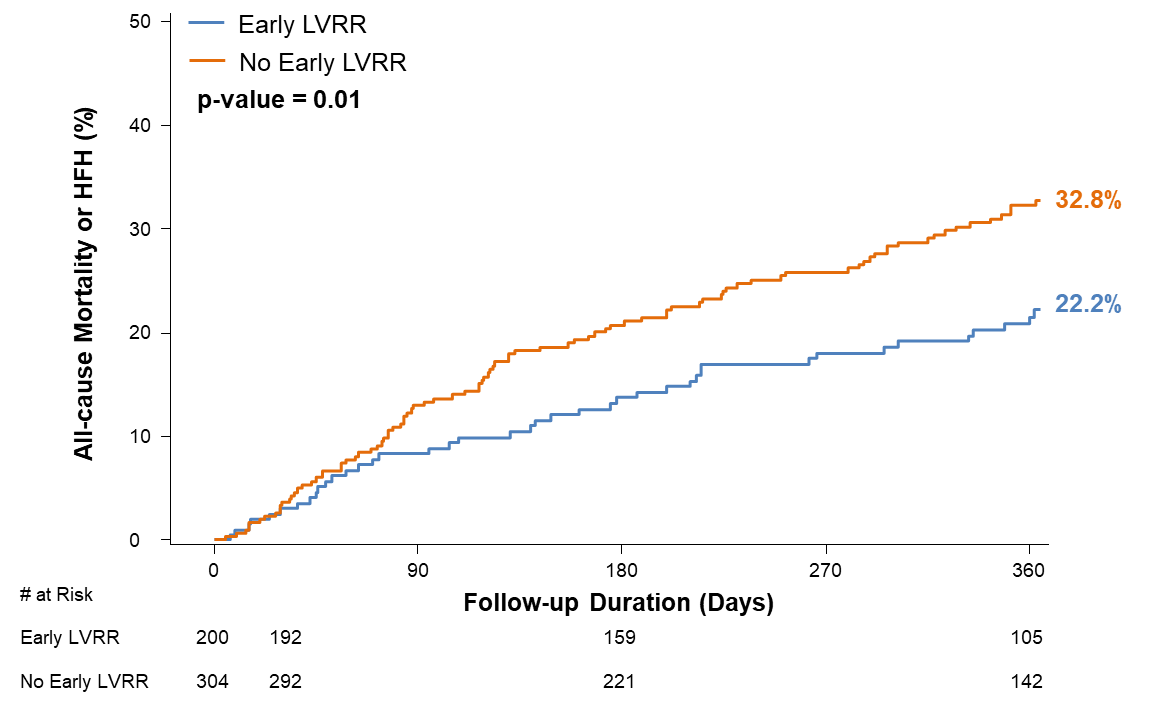


**Supplemental Figure 4. Composite all-cause mortality or heart failure hospitalization (HFH) through 1 year in patients with secondary mitral regurgitation (MR) with a volume anchored and stricter definition for early LVRR.** Kaplan-Meier estimate for patients with primary MR and either early left ventricular reverse remodeling (LVRR, blue) or no early LVRR (orange). Significance by log-rank test.

**
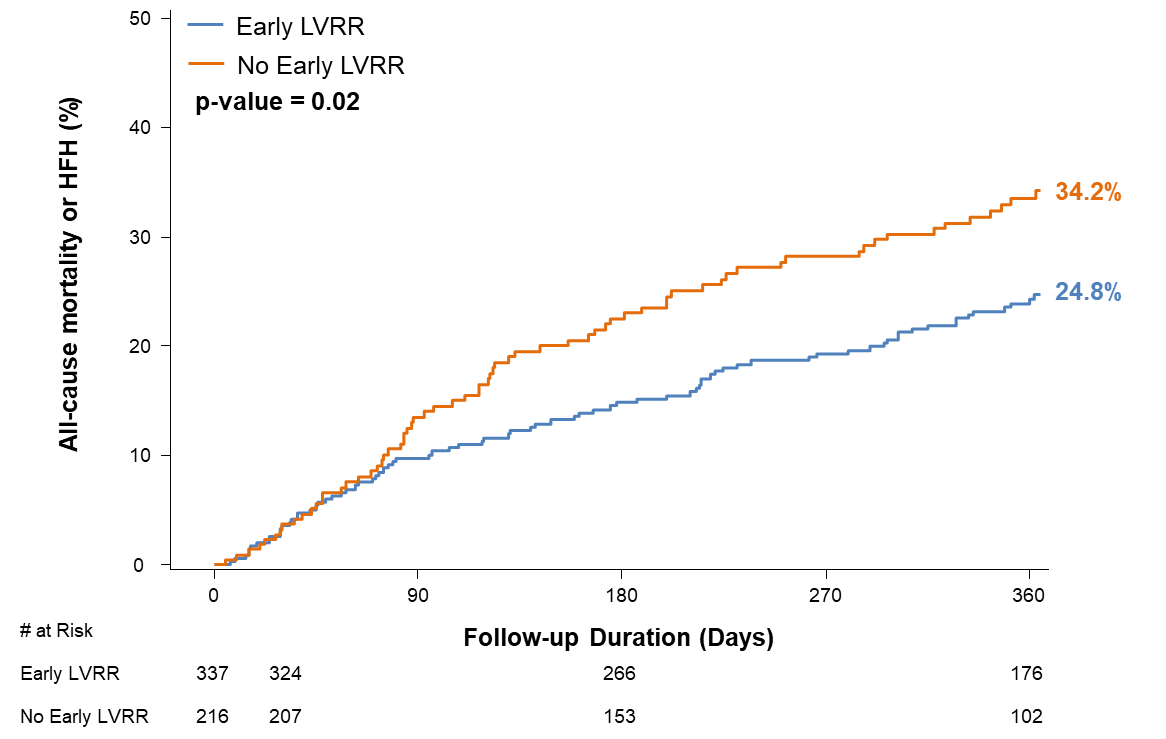
**

**Supplemental Figure 5. Composite all-cause mortality or heart failure hospitalization (HFH) through 1 year in patients with secondary mitral regurgitation (MR) applying the LVRR definition with indexed LV dimensions and volumes (indexed by body surface area).** Kaplan-Meier estimate for patients with primary MR and either early left ventricular reverse remodeling (LVRR, blue) or no early LVRR (orange). Significance by log-rank test.


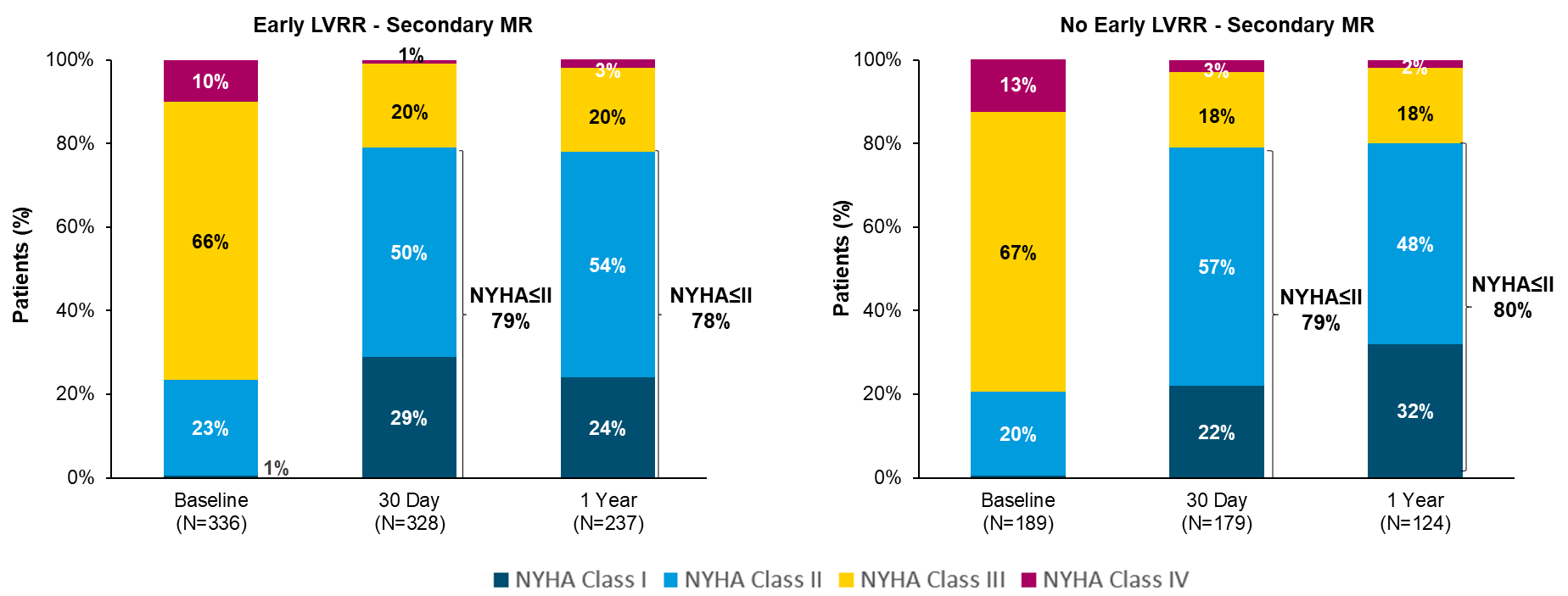


**
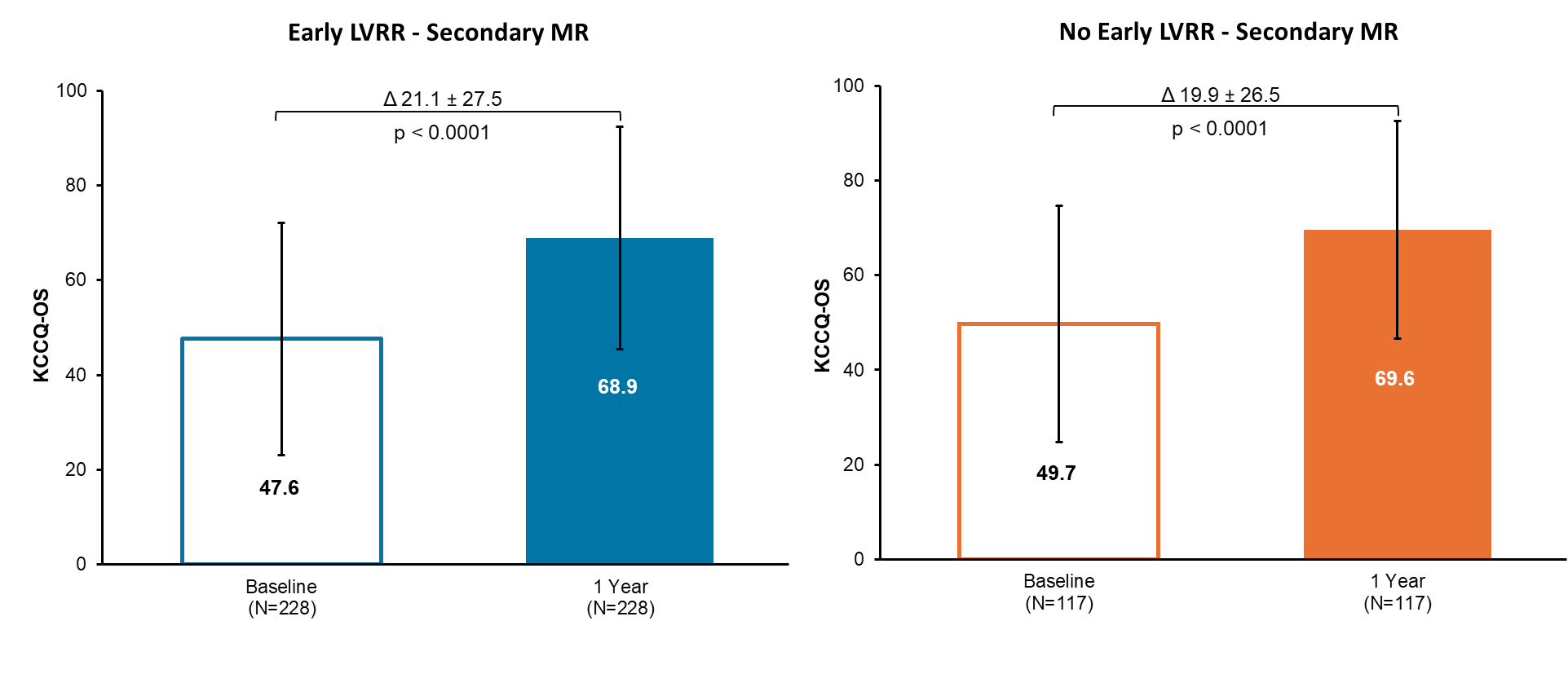
**


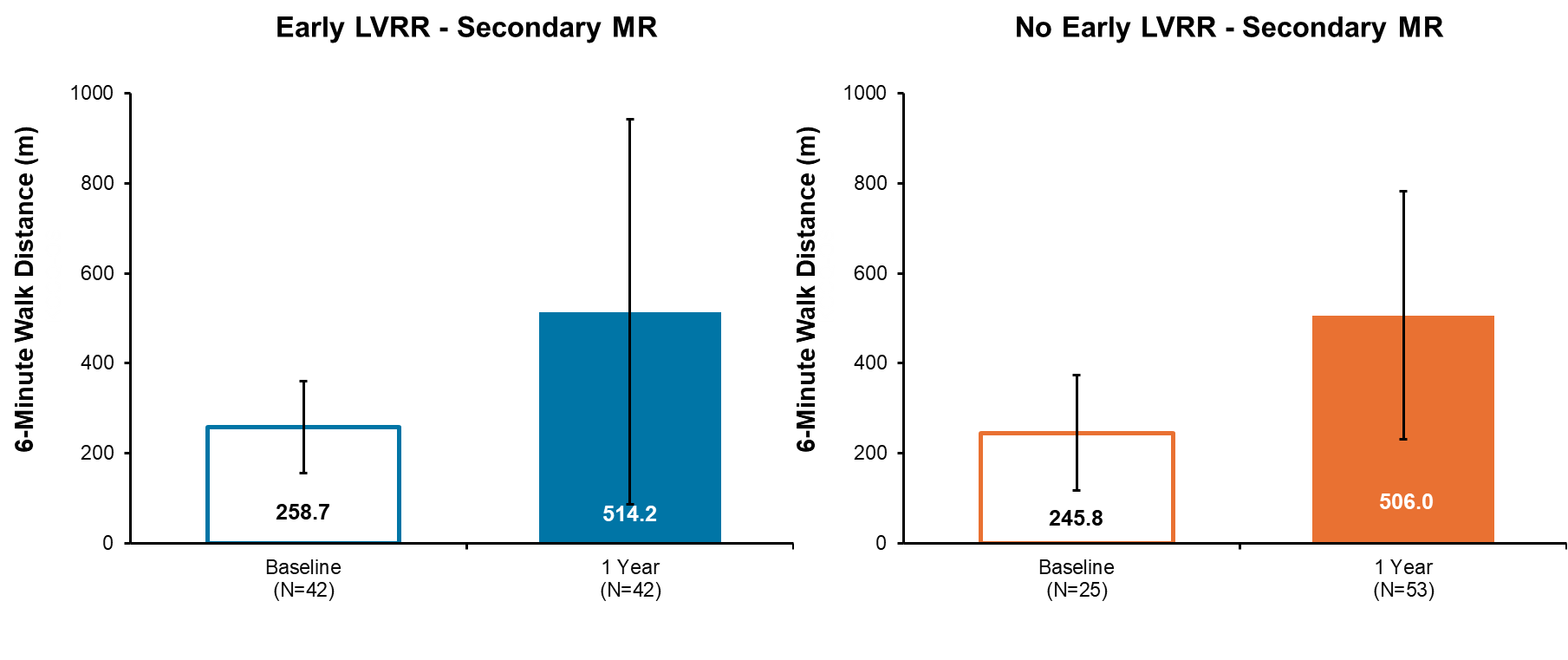
**Supplemental Figure 6. Functional and symptomatic outcomes through 1 year in patients with secondary mitral regurgitation (MR).** New York Heart Association (NYHA) Class (top), Kansas City Cardiomyopathy Questionnaire overall summary (KCCQ-OS) score (middle, Significance by Student’s t-test), and six-minute walk distance in patients with secondary MR and early left ventricular reverse remodeling (LVRR, left) or no early LVRR (right).

**
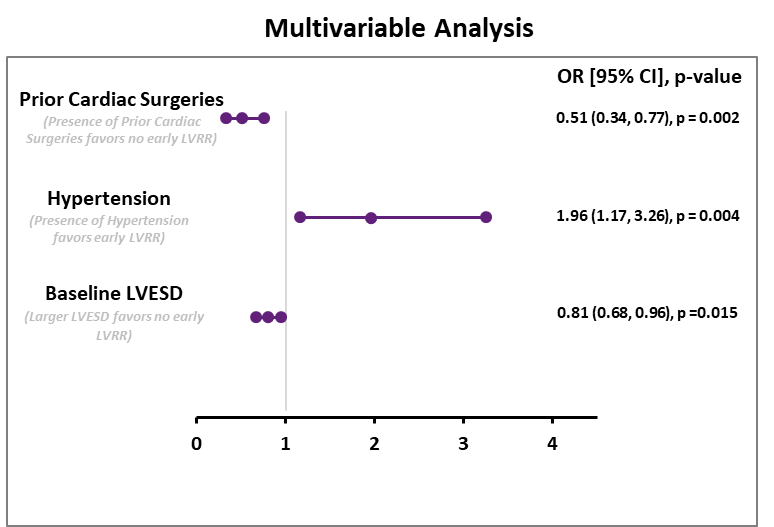
**

**Supplemental Figure 7. Associations with early left ventricular reverse remodeling (LVRR) in patients with secondary mitral regurgitation.** Multivariable analysis of associations with early LVRR in patients with secondary mitral regurgitation indicated hypertension favored early LVRR while prior cardiac surgeries and larger baseline left ventricular end-systolic diameter (LVESD) favored no early LVRR.

**
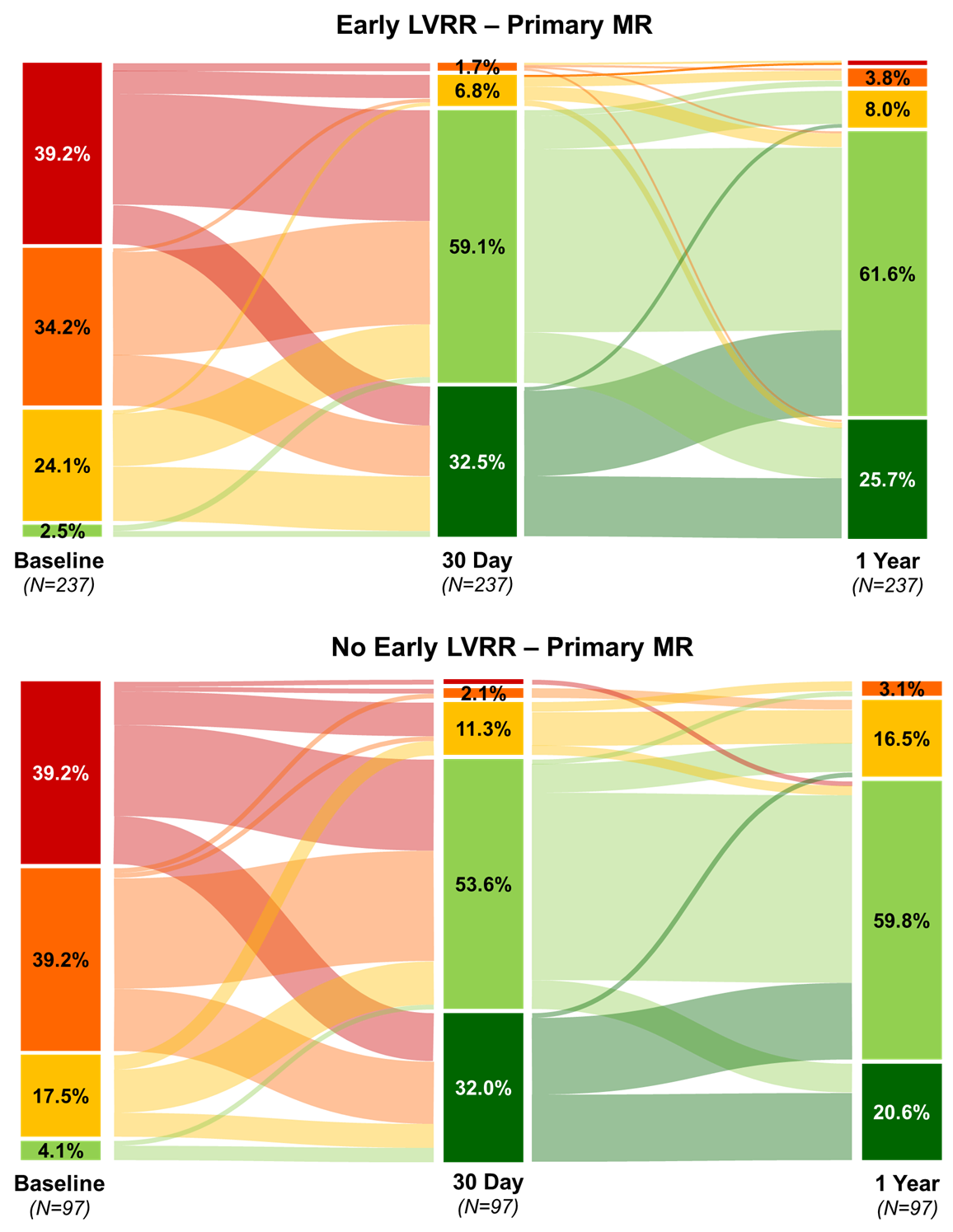
**

**Supplemental Figure 8.** **Paired mitral regurgitation (MR) severity through 1 year by early left ventricular reverse remodeling (LVRR) in patients with primary MR.**

**
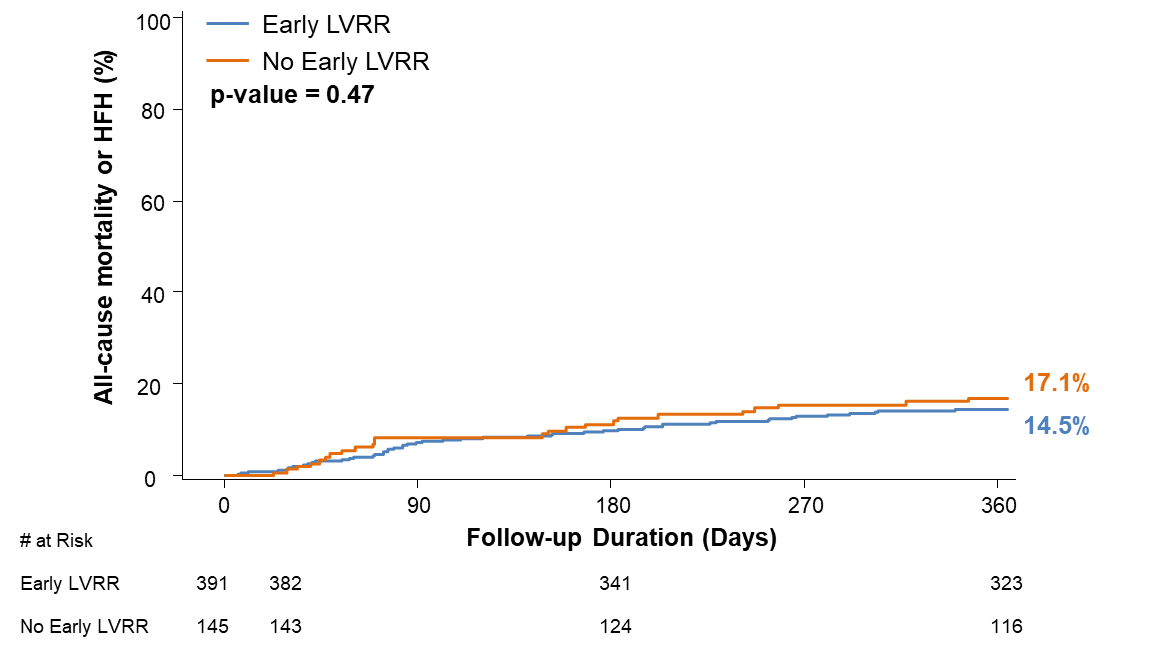
**

**Supplemental Figure 9. Composite all-cause mortality or heart failure hospitalization (HFH) through 1 year in patients with primary mitral regurgitation (MR).** Kaplan-Meier estimate for patients with primary MR and either early left ventricular reverse remodeling (LVRR, blue) or no early LVRR (orange). Significance by log-rank test.

**
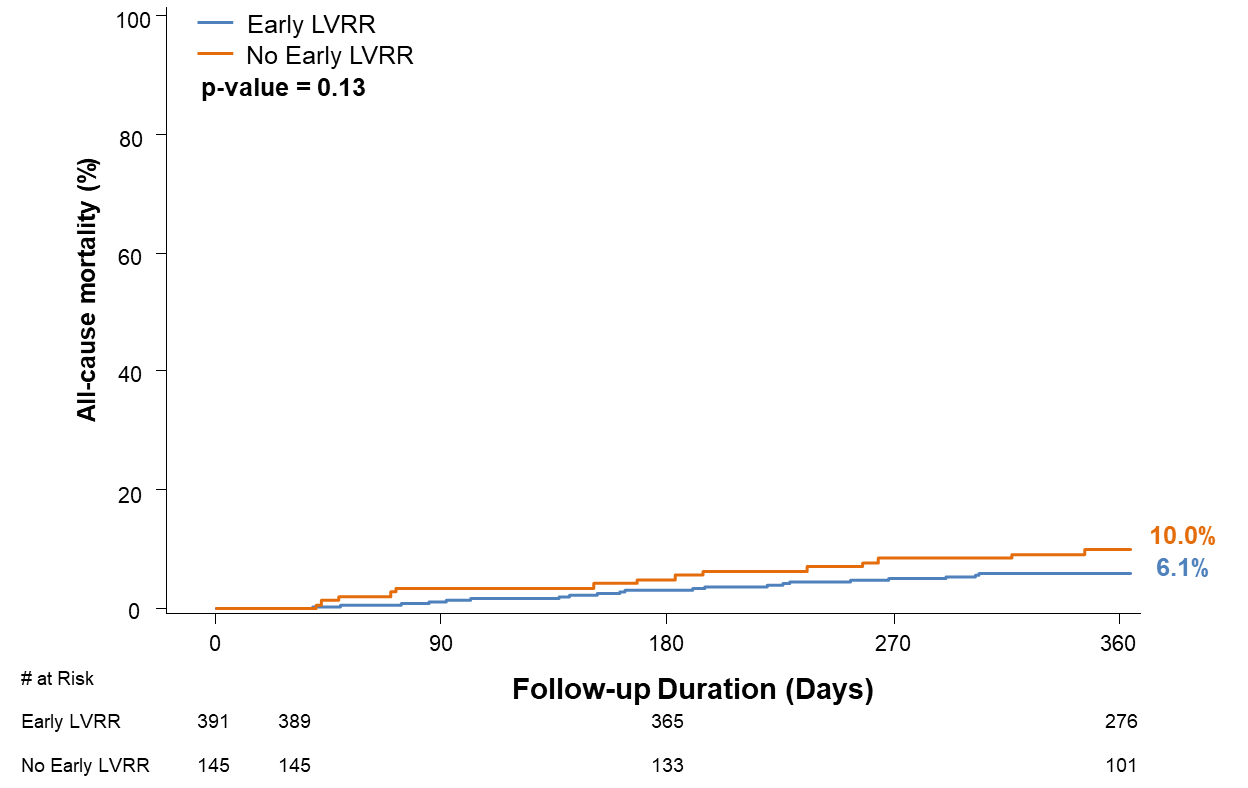

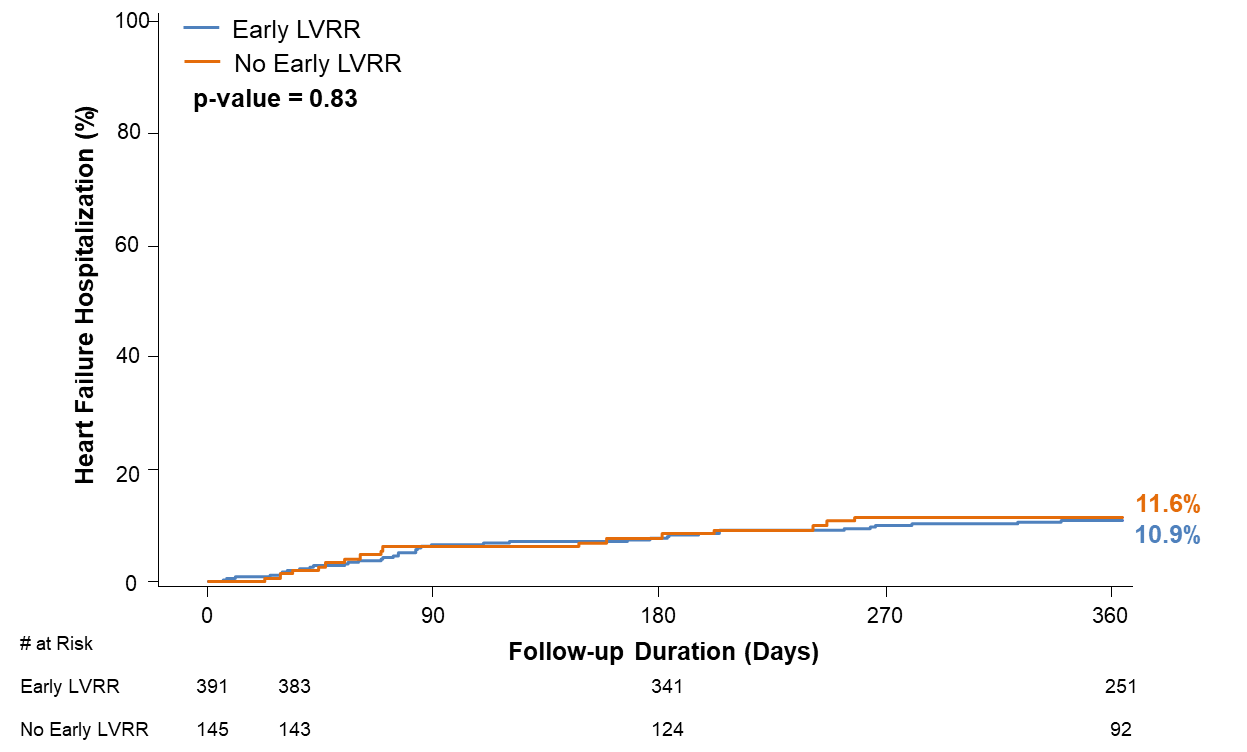
**

**Supplemental Figure 10. All-cause mortality and heart failure hospitalization (HFH) through 1 year in patients with primary mitral regurgitation.** Kaplan-Meier estimates for early (blue) or no early left ventricular reverse remodeling (LVRR, orange) groups. Significance by log-rank test.

**
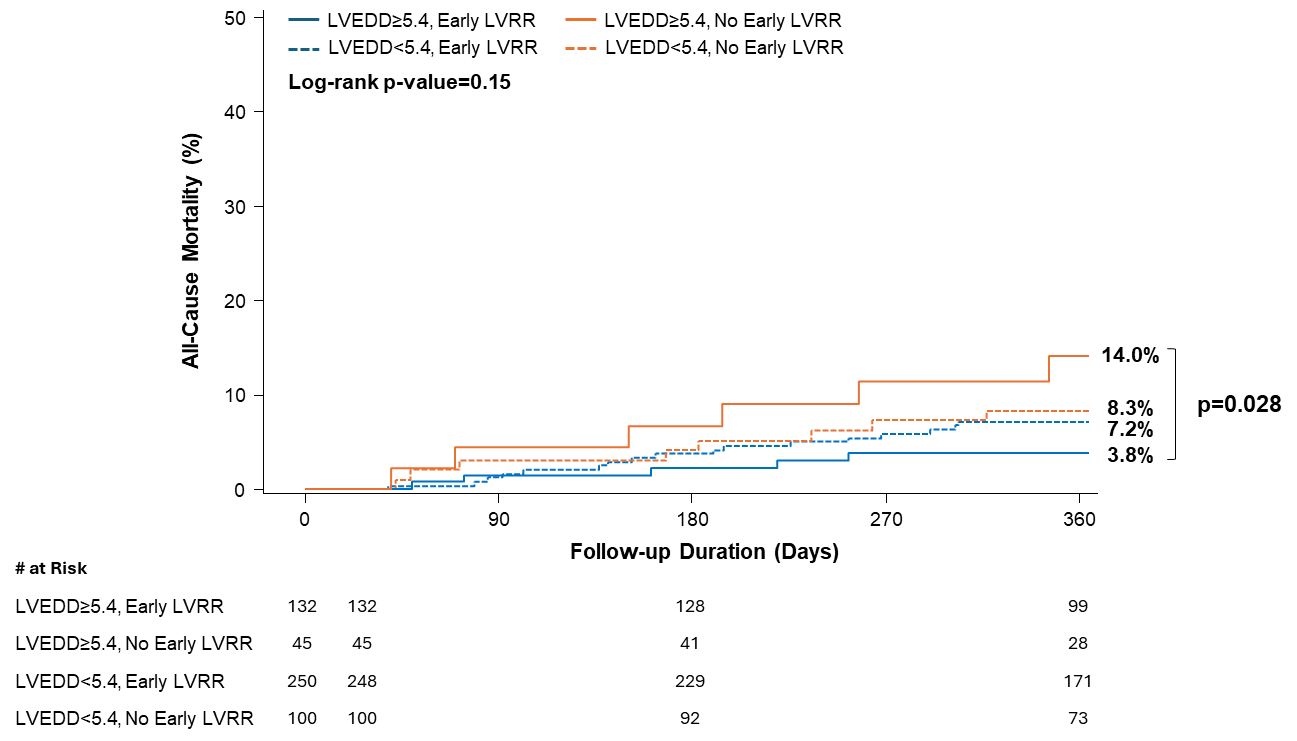
**

**Supplemental Figure 11. All-cause mortality by baseline left ventricular end diastolic dimension (LVEDD) and presence of early or not early left ventricular reverse remodeling (LVRR) in patients with primary mitral regurgitation.** Significance by log-rank test.

**
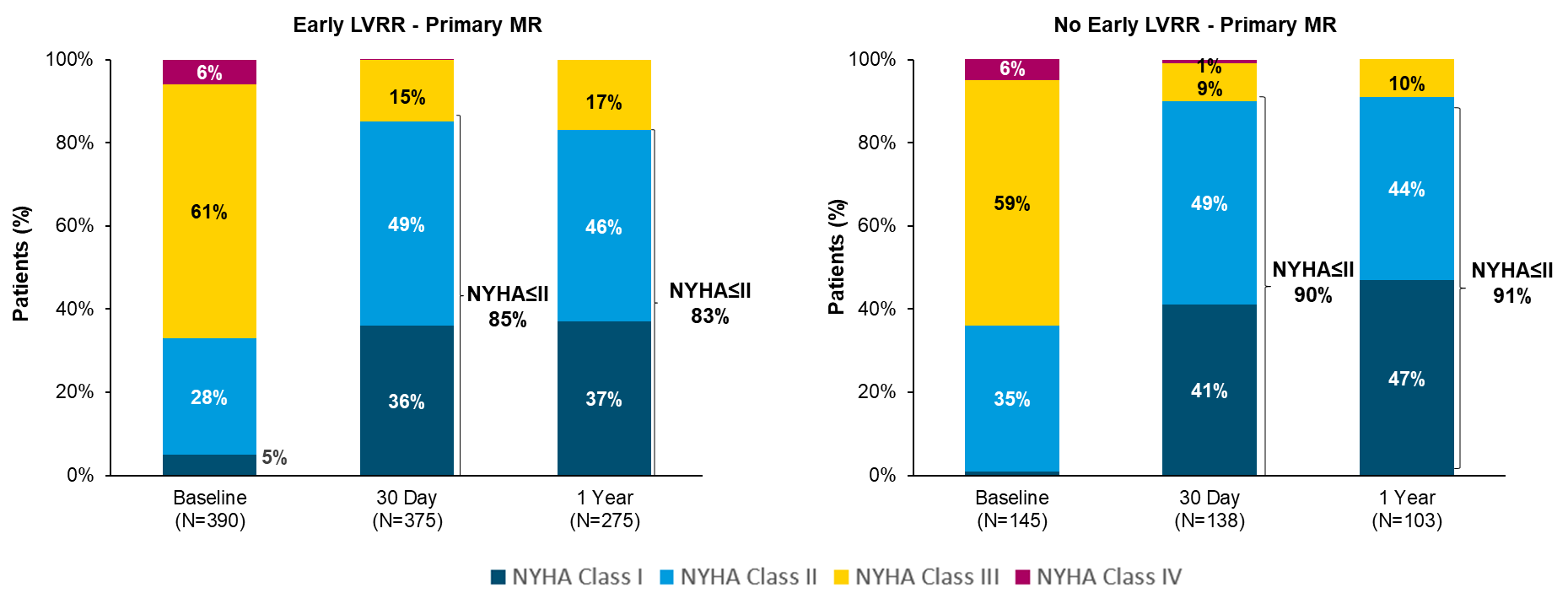
**


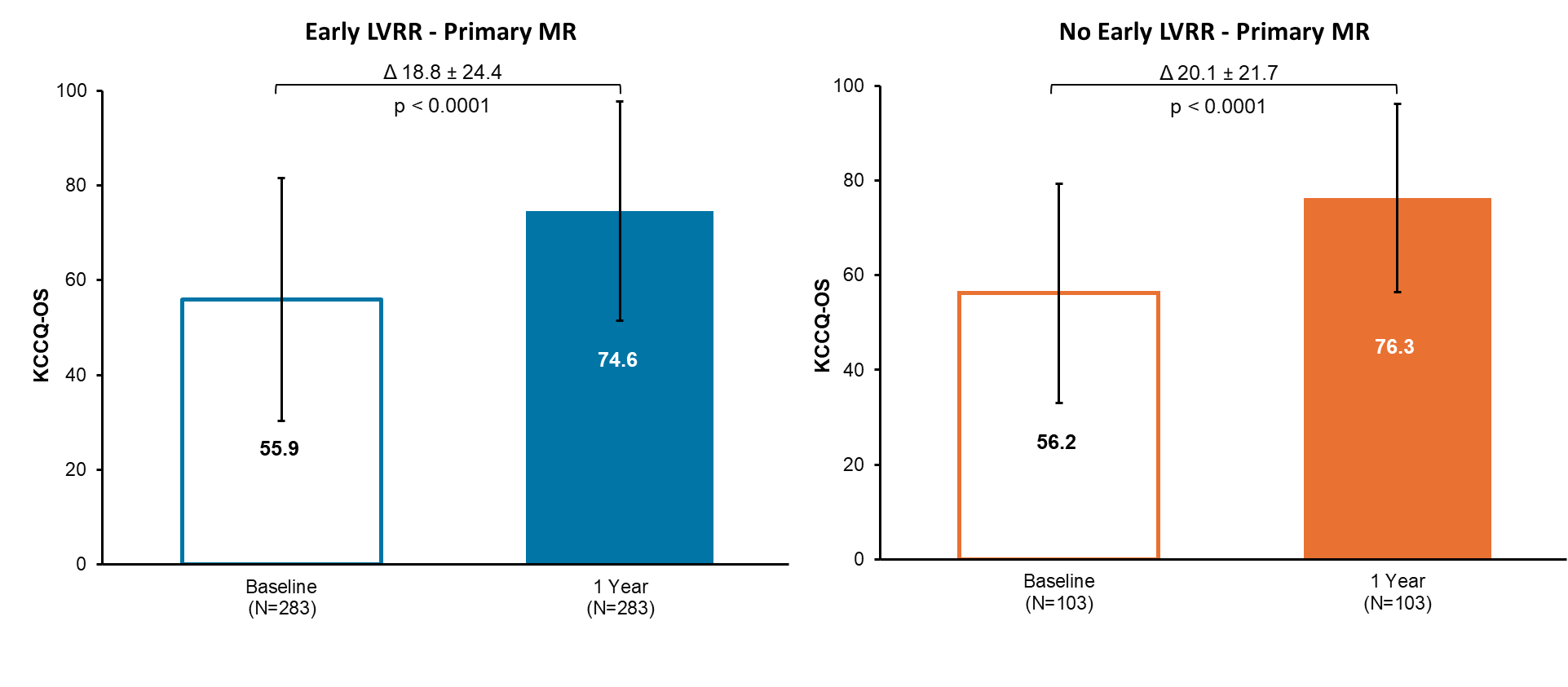

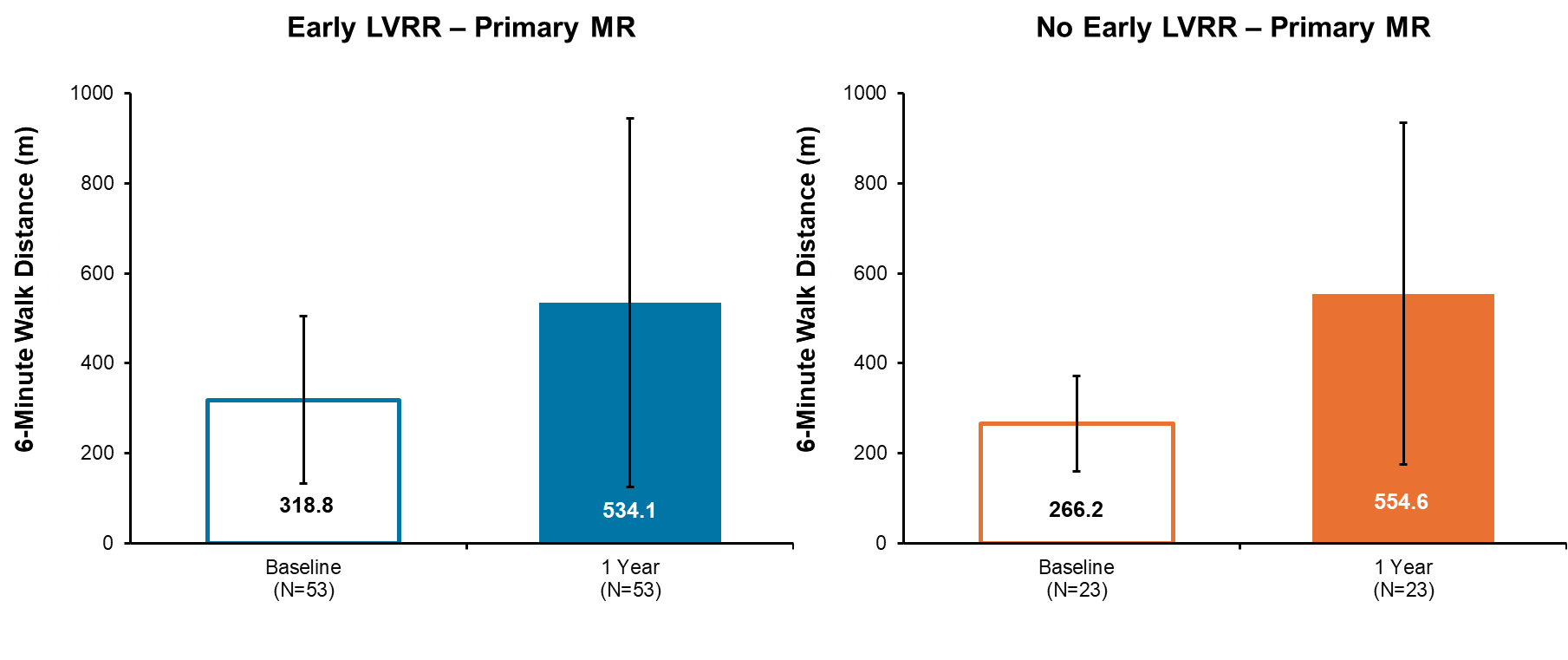
**Supplemental Figure 12. Functional and symptomatic outcomes through 1 year in patients with primary mitral regurgitation (MR).** New York Heart Association (NYHA) Class (top), Kansas City Cardiomyopathy Questionnaire overall summary (KCCQ-OS) score (middle, Significance by Student’s t-test), and six-minute walk distance in patients with primary MR and early left ventricular reverse remodeling (LVRR, left) or no early LVRR (right).

**Supplemental Table 1**. Baseline characteristics of patients excluded from analysis due to missing paired left ventricular (LV) measurements.

| **Variables** | **Excluded Cohort (N=1045)** | **Full Cohort (N=1194)** | **P Value** |
| --- | --- | --- | --- |
| **Age** | 77.57 ± 9.32 | 77.21 ± 9.45 | 0.23 |
| Female | 45.3% (473) | 44.1% (527) | 0.59 |
| STS Repair Score | 6.42 ± 6.82 | 5.73 ± 5.65 | 0.16 |
| Hypertension | 80.3% (830) | 80.5% (956) | 0.94 |
| Atrial Fibrillation | 61.0% (630) | 56.9% (676) | 0.05 |
| Prior Myocardial Infarction | 23.6% (238) | 20.2% (238) | 0.06 |
| Prior HFH within 1 year | 47.2% (447) | 48.8% (537) | 0.45 |
| Left Ventricular Ejection Fraction (LVEF, %) | 48.31 ± 16.77 | 50.69 ± 15.91 | **0.01** |
| Left Ventricular End Systolic Volume (LVESV, mL) | 79.22 ± 57.13 | 78.21 ± 58.73 | 0.77 |
| Indexed LVESV | 43.61 ± 30.99 | 42.30 ± 30.65 | 0.63 |
| Left Ventricular End Diastolic Volume (LVEDV, mL) | 140.94 ± 64.96 | 145.99 ± 68.39 | 0.24 |
| Indexed LVEDV | 77.90 ± 34.15 | 79.30 ± 34.32 | 0.51 |
| Left Ventricular End Systolic Dimension (LVESD, cm) | 4.19 ± 1.21 | 4.17 ± 1.19 | 0.78 |
| Left Ventricular End Diastolic Dimension (LVEDD, cm) | 5.47 ± 0.95 | 5.54 ± 0.91 | 0.11 |
| Left Atrial Volume | 103.45 ± 57.25 | 104.20 ± 56.65 | 0.39 |

**Supplemental Table 2.** Baseline medication usage in patients with secondary mitral regurgitation.

| **Baseline Medications** | **Early LVRR** | **No Early LVRR** | **p-values** |
| --- | --- | --- | --- |
| Any heart failure medications | 97.9 (331/338) | 99.5 (188/189) | 0.17 |
| ACE-Inhibitors (ACE-I) | 29.6 (100/338) | 26.5 (50/189) | 0.45 |
| Aldosterone Antagonists | 27.2 (92/338) | 36.0 (68/189) | **0.04** |
| Angiotensin Receptor Blockers (ARB) | 21.6 (73/338) | 18.0 (34/189) | 0.32 |
| Beta-Blockers | 82.2 (278/338) | 88.9 (168/189) | **0.04** |
| Digitalis | 7.7 (26/338) | 5.8 (11/189) | 0.42 |
| Diuretics | 81.4 (275/338) | 88.9 (168/189) | **0.02** |
| Entresto | 16 (54/338) | 21.2 (40/189) | 0.14 |
| Vasodilators (Nitrate and Hydralazine) | 11.5 (39/338) | 12.7 (24/189) | 0.69 |
| Anti-coagulants | 53.6 (181/338) | 52.4 (99/189) | 0.80 |
| Warfarin | 12.7 (43/338) | 17.5 (33/189) | 0.14 |
| NOAC | 40.5 (137/338) | 34.9 (66/189) | 0.20 |
| Anti-plateles | 55.6 (188/338) | 54 (102/189) | 0.71 |
| Aspirin | 43.8 (148/338) | 49.7 (94/189) | 0.19 |
| P2Y12 antagonists | 23.7 (80/338) | 19.6 (37/189) | 0.28 |
| Statins | 68.6 (232/338) | 70.9 (134/189) | 0.59 |
| Cardiac medications | 19.2 (65/338) | 27 (51/189) | **0.04** |
| Antiarrhythmic | 16.6 (56/338) | 22.8 (43/189) | 0.08 |
| Positive inotropic agents | 3.8 (13/338) | 5.3 (10/189) | 0.44 |

LVRR, left ventricular reverse remodeling; NOAC, non-oral anticoagulation.

**Supplemental Table 3. Univariable analysis with early LVRR in patients with secondary mitral regurgitation (MR).**

| **Parameter** | **Unadjusted OR**  **(95% CI)** | **P-value** |
| --- | --- | --- |
| Age | 1.01 (0.99, 1.03) | 0.38 |
| Female | 1.29 (0.89, 1.86) | 0.17 |
| STS Score - Mitral Valve Replacement | 1.01 (0.97, 1.05) | 0.69 |
| STS Score - Mitral Valve Repair | 0.99 (0.96, 1.03) | 0.70 |
| Diabetes | 1.19 (0.81, 1.75) | 0.38 |
| Renal Failure | 0.94 (0.65, 1.36) | 0.74 |
| Chronic lung disease | 1.03 (0.68, 1.57) | 0.89 |
| Chronic obstructive pulmonary disease | 1.22 (0.76, 1.96) | 0.40 |
| Prior cardiac surgeries | 0.53 (0.37, 0.77) | **0.001** |
| Prior heart failure hospitalization within 1 year | 0.80 (0.55, 1.17) | 0.26 |
| Hypertension | 1.84 (1.16, 2.91) | **0.01** |
| Home Oxygen | 2.16 (0.79, 5.92) | 0.13 |
| Peripheral arterial disease | 1.18 (0.68, 2.05) | 0.57 |
| NYHA III/IV | 0.85 (0.55, 1.30) | 0.45 |
| CRT/CRT-D/ICD/Permanent Pacemaker | 0.54 (0.36, 0.81) | **0.003** |
| Baseline MR≥2+ | 1.25 (0.60, 2.58) | 0.55 |
| Baseline MR≥3+ | 1.04 (0.73, 1.50) | 0.82 |
| Baseline TR≥2+ | 0.85 (0.58, 1.23) | 0.39 |
| Septal-Lateral Mitral Valve Annulus Diastole (cm) | 0.82 (0.53, 1.29) | 0.39 |
| Anterior-Posterior Diastolic Annular Dimension (cm) | 1.19 (0.82, 1.75) | 0.36 |
| Effective Regurgitant Orifice Area (cm^2^) | 0.55 (0.10, 3.18) | 0.51 |
| Baseline Mean Mitral Gradient (mmHg) | 0.97 (0.81, 1.17) | 0.77 |
| Left Ventricular Ejection Fraction | 1.02 (1.01, 1.03) | **0.006** |
| Left Ventricular End Systolic Volume (ml) | 0.96 (0.94, 0.99) | **0.006** |
| Left Ventricular End Diastolic Volume (ml) | 0.97 (0.95, 1.00) | **0.02** |
| Left Ventricular End Diastolic Dimension | 0.75 (0.62, 0.91) | **0.004** |
| Left Ventricular End Systolic Dimension (cm) | 0.77 (0.65, 0.90) | **<0.001** |
| Systolic Pulmonary Artery Pressure (mmHg) | 1.00 (0.99, 1.01) | 0.76 |
| Baseline Mitral Valve Area (cm^2^) | 1.03 (0.90, 1.18) | 0.69 |
| Mitral Valve Peak E Velocity (cm/s) | 1.01 (0.94, 1.09) | 0.76 |
| Coaptation Length (cm) | 2.18 (0.61, 7.80) | 0.23 |
| Total Number of Clips | 0.81 (0.61, 1.09) | 0.16 |
| Discharge Anterior Posterior Diastolic Annular Dimension (cm) | 0.73 (0.51, 1.04) | 0.08 |
| Discharge Mitral Valve Area (cm^2^) | 0.81 (0.61, 1.06) | 0.12 |
| Discharge Mean Gradient (mmHg) | 0.98 (0.92, 1.04) | 0.50 |
| MR≥2+ at Discharge | 0.91 (0.42, 1.98) | 0.82 |

LVRR, left ventricular reverse remodeling; MR, mitral regurgitation; OR, odds ratio; STS, Society of Thoracic Surgeons; TR, tricuspid regurgitation.

**Supplemental Table 4. Univariable analysis with early LVRR in patients with primary mitral regurgitation.**

| **Parameter** | **Unadjusted OR (95% CI)** | **P-value** |
| --- | --- | --- |
| Age | 0.99 (0.97, 1.02) | 0.56 |
| Female | 0.84 (0.57, 1.23) | 0.37 |
| STS Score - Mitral Valve Replacement | 0.99 (0.95, 1.04) | 0.76 |
| STS Score - Mitral Valve Repair | 0.97 (0.93, 1.02) | 0.26 |
| Diabetes | 0.98 (0.58, 1.63) | 0.92 |
| Renal Failure | 1.29 (0.80, 2.09) | 0.30 |
| Chronic lung disease | 0.88 (0.53, 1.44) | 0.61 |
| Chronic obstructive pulmonary disease | 1.03 (0.57, 1.87) | 0.91 |
| Prior cardiac surgeries | 0.87 (0.53, 1.43) | 0.59 |
| Prior heart failure hospitalization within 1 year | 1.09 (0.72, 1.64) | 0.70 |
| Hypertension | 0.74 (0.46, 1.19) | 0.21 |
| Home Oxygen | 0.89 (0.31, 2.57) | 0.83 |
| Peripheral arterial disease | 0.83 (0.43, 1.62) | 0.59 |
| NYHA III/IV | 1.14 (0.77, 1.71) | 0.51 |
| CRT/CRT-D/ICD/Permanent Pacemaker | 2.00 (0.67, 5.93) | 0.21 |
| Baseline MR≥2+ | 0.89 (0.32, 2.50) | 0.83 |
| Baseline MR≥3+ | 0.71 (0.46, 1.11) | 0.14 |
| Baseline TR≥2+ | 1.00 (0.67, 1.51) | 0.98 |
| Septal-Lateral Mitral Valve Annulus Diastole (cm) | 1.02 (0.65, 1.60) | 0.94 |
| Anterior-Posterior Diastolic Annular Dimension (cm) | 1.33 (0.92, 1.93) | 0.13 |
| Effective Regurgitant Orifice Area (cm^2^) | 1.26 (0.86, 1.85) | 0.24 |
| Baseline Mean Mitral Gradient (mmHg) | 1.98 (0.57, 6.84) | 0.28 |
| Left Ventricular Ejection Fraction | 1.06 (0.90, 1.24) | 0.49 |
| Left Ventricular End Systolic Volume (ml) | 1.01 (0.99, 1.03) | 0.45 |
| Left Ventricular End Diastolic Volume (ml) | 1.04 (0.96, 1.11) | 0.35 |
| Left Ventricular End Diastolic Dimension | 1.06 (1.01, 1.11) | 0.01 |
| Left Ventricular End Systolic Dimension (cm) | 1.12 (0.85, 1.48) | 0.41 |
| Systolic Pulmonary Artery Pressure (mmHg) | 1.29 (1.00, 1.66) | 0.05 |
| Baseline Mitral Valve Area (cm^2^) | 1.01 (1.00, 1.02) | 0.21 |
| Mitral Valve Peak E Velocity (cm/s) | 0.91 (0.78, 1.06) | 0.22 |
| Coaptation Length (cm) | 1.03 (0.97, 1.10) | 0.35 |
| Total Number of Clips | 3.44 (0.54, 21.95) | 0.19 |
| Discharge Anterior Posterior Diastolic Annular Dimension (cm) | 0.91 (0.67, 1.24) | 0.55 |
| Discharge Mitral Valve Area (cm^2^) | 1.10 (0.78, 1.57) | 0.59 |
| Discharge Mean Gradient (mmHg) | 1.05 (0.72, 1.54) | 0.79 |
| MR≥2+ at Discharge | 1.03 (0.95, 1.11) | 0.47 |
| MR≥2+ (Discharge) | 0.90 (0.50, 1.62) | 0.73 |
| MR≥3+ (Discharge) | 0.29 (0.08, 1.10) | 0.07 |

LVRR, left ventricular reverse remodeling; MR, mitral regurgitation; OR, odds ratio; STS, Society of Thoracic Surgeons; TR, tricuspid regurgitation.
